# A glutamate-gated chloride channel as the mite-specific target-site of dicofol and other diphenylcarbinol acaricides

**DOI:** 10.1038/s42003-023-05488-5

**Published:** 2023-11-13

**Authors:** Marilou Vandenhole, Catherine Mermans, Berdien De Beer, Wenxin Xue, Yilan Zhao, Yoshihisa Ozoe, Genyan Liu, Wannes Dermauw, Thomas Van Leeuwen

**Affiliations:** 1 Department of Plants and Crops, Faculty of Bioscience Engineering, Ghent University, Coupure links 653, Ghent, Belgium; 2 School of Chemical Engineering and Pharmacy, Wuhan Institute of Technology, 693 Xiongchu Blvd, Wuhan, China; 3 Faculty of Life and Environmental Science, Shimane University, Matsue, Japan; 4 Flanders Research Institute for Agriculture, Fisheries and Food (ILVO), Plant Sciences Unit, Burgemeester Van Gansberghelaan 96, Merelbeke, Belgium

**Keywords:** Entomology, Agricultural genetics, Chloride channels

## Abstract

Dicofol has been widely used to control phytophagous mites. Although dicofol is chemically related to DDT, its mode of action has remained elusive. Here, we mapped dicofol resistance in the spider mite *Tetranychus urticae* to two genomic regions. Each region harbored a glutamate-gated chloride channel (GluCl) gene that contained a mutation—G314D or G326E—known to confer resistance against the unrelated acaricide abamectin. Using electrophysiology assays we showed that dicofol and other diphenylcarbinol acaricides—bromopropylate and chlorobenzilate—induce persistent currents in *Xenopus* oocytes expressing wild-type *T. urticae* GluCl3 receptors and potentiate glutamate responses. In contrast, the G326E substitution abolished the agonistic activity of all three compounds. Assays with the wild-type *Drosophila* GluClα revealed that this receptor was unresponsive to dicofol. Homology modeling combined with ligand-docking confirmed the specificity of electrophysiology assays. Altogether, this work elucidates the mode of action of diphenylcarbinols as mite-specific agonists of GluCl.

## Abstract (web summary)

Mapping dicofol resistance in the spider mite *Tetranychus urticae* shows that diphenylcarbinols like dicofol act as mite-specific agonists of glutamate-gated chloride channels, providing valuable insights for selective pest control strategies.

## Introduction

Dicofol (2,2,2-trichloro-1,1-bis(4-chlorophenyl)ethanol) is a selective acaricide that was introduced commercially in 1955^1^ and has been widely used in many regions of the world since the early 70s^2^. It is structurally related to the organochlorine insecticide DDT (dichlorodiphenyltrichloroethane) which acts as a modulator of the voltage-gated sodium channel (VGSC) and has historically been of great importance in the control of many insect pests that attack our crops or threaten animal and human health^3,4^. However, this broad spectrum pesticide and its main metabolites DDE (dichlorodiphenyldichloroethylene) and DDD (tetrachlorodiphenylethane) were banned in many regions due to the lack of selectivity, environmental persistence and capacity to accumulate in adipose tissues^5,6^. Remarkably, DDT is still being used in African and Asian regions, mostly via indoor residual spraying, to control mosquitoes that transmit vector-borne diseases^7^.

**Fig. 1:**
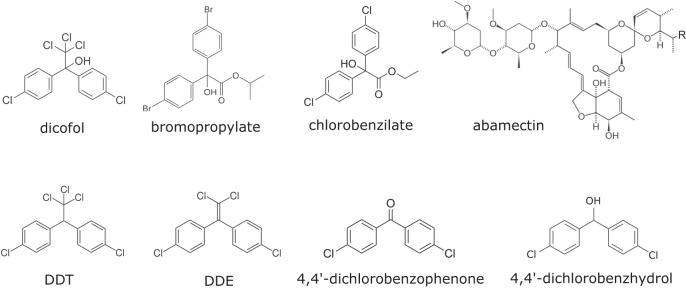
Chemical structures. Overview of chemical structures of dicofol, bromopropylate, chlorobenzilate, DDT, DDE, abamectin, 4,4′-dichlorobenzophenone and 4,4′ dichlorobenzhydrol.

In contrast to DDT, dicofol and other diphenylcarbinols such as chlorobenzilate and bromopropylate, are very well known for their excellent and specific activity on mite species^1,8^. The strong difference in selectivity between DDT and dicofol could be due to a different mode of action, even though dicofol and DDT only differ in structure by a single hydroxyl-group (Fig. 1). Octopamine-stimulated adenylate cyclase, Mg^2+^-dependent ATPases, and Na^+^, K^+^ ATPase have all been suggested as potential target-sites for dicofol^1,9–15^ but, despite its use for more than 60 years, the actual target-site of dicofol has remained elusive^16^. This is surprising, as understanding the mode of action of pesticides is crucial for implementing resistance management strategies and developing molecular diagnostic tools, but also for the rational design of new resistance breaking compounds^16,17^.

*Tetranychus urticae* is an extremely polyphagous pest that can thrive on more than 1000 plant species, including many agricultural crops. It is notorious for its ability to quickly develop resistance due to its high fecundity, rapid development, and arrhenotokous reproduction^2,18^. Not surprisingly, because of its frequent use, dicofol resistance has evolved in the field and over 25 cases of dicofol resistance have been described and investigated thus far^3,10,19–22^. Resistance mechanisms in arthropods comprise both toxicodynamic and toxicokinetic mechanisms. In case of the latter, the exposure to toxic substances is decreased either by reduced uptake, increased metabolic detoxification or sequestration and excretion. Toxicodynamic changes, on the other hand, alter the target-site, resulting in decreased sensitivity towards acaricides^4,18^. Next to this classical distinction, an alternative classification of resistance mechanisms—based on genotypic changes—has also been proposed (but see Feyereisen et al.^23^ for more details). Given that the target-site of dicofol is currently unknown, dicofol resistance has mainly been linked with increased metabolic detoxification^10,24,25^. For example, Tabata & Saito showed that resistance to dicofol was associated with increased detoxification to aqueous metabolites^25,26^. However, Kim et al. showed that inhibitors of detoxification enzymes were not able to synergize dicofol toxicity in a 400-fold dicofol resistant spider mite strain, suggesting an altered target-site as a likely mechanism^12^. Of important note, despite the fact that dicofol has not been used in Europe for a decade, populations with high levels of resistance are still encountered today^27^, which might indicate that resistance genes are retained in the population by selection of other acaricides.

In this study, we use highly inbred lines of *T. urticae*^27^, together with experimental evolution and bulked segregant analysis (BSA), to map genomic regions (QTLs) associated with dicofol resistance in the spider mite *T. urticae*. This species is especially suitable for a BSA-based QTL mapping approach due to the ease of mite husbandry, its short life cycle and arrhenotokous reproduction (see Kurlovs et al.^28^ for a review). Genome scans revealed two genomic loci that both harbored a glutamate-gated chloride channel (GluCl) gene, with both GluCl genes carrying a mutation that was previously reported to confer resistance against the unrelated acaricide abamectin. The role of these mutations in resistance against dicofol and other diphenylcarbinol acaricides was further validated with two-electrode voltage clamp electrophysiology (TEVC) assays, homology modeling, and ligand docking.

## Results

### Bulked segregant analysis

#### Dicofol toxicity in parental strains and BSA experimental populations

**Fig. 2:**
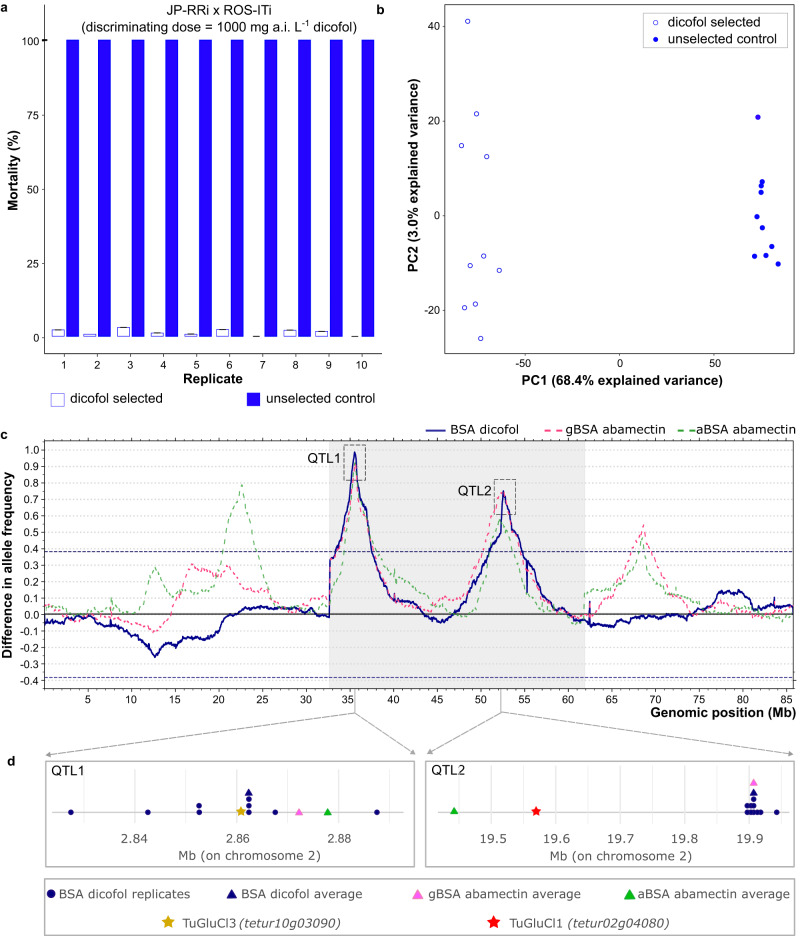
Phenotypic and genomic responses to dicofol selection. **a** Adult corrected mortality of long-term selected and control (unselected) populations after application of 1000 mg L^-1^ dicofol. Unselected populations showed significantly higher mortality rates compared to the control populations (F_1,74_ = 78472, *p* < 2.2 e^-16^). Error bars represent standard error of the mean (*n* = 4). **b** Principal component analysis (PCA) of the unselected and dicofol selected populations of JP-RRi x ROS-ITi, based on genome-wide allele frequencies at polymorphic sites. PC1 clearly separates the selected populations from the unselected populations. **c** Overlay of QTL mapping for resistance to dicofol with bulked segregant analysis (BSA) (blue, solid line) in this study and two BSA scans for abamectin selection, gBSA (abamectin BSA, conducted at Ghent University, starting from cross between susceptible JP-RRi and resistant ROS-ITi strains) and aBSA (abamectin BSA, conducted at University of Amsterdam, starting from cross between susceptible SOL-BEi and resistant MAR-ABi strains) (pink and green dashed lines respectively) from data published in the study of Villacis-Perez et al. (2023)^32^. Scans reflect averaged genome-wide differences in allele frequency using ten paired populations (dicofol selected vs unselected). For dicofol, two QTLs on chromosome 2 exceeded the 5% FDR threshold (dashed blue lines delineate the statistical significance for QTL detection): QTL1 ( ~ 2.862 Mb) and QTL2 ( ~ 19.907 Mb). **d** The chromosomal location of QTL1 (left) and QTL2 (right) for each replicate of the dicofol BSA is represented by a solid blue circle. The chromosomal location of peak averages calculated by combining all replicates into a single analysis for dicofol BSA, gBSA and aBSA is represented by a solid blue, pink and green triangle, respectively. The locations of GluCl genes TuGluCl3 (*tetur10g03090*) and TuGluCl1 (*tetur02g04080*) are indicated with a solid brown or red star, respectively.

To uncover the genomic loci involved in dicofol resistance, a bulked segregant analysis was performed. Toxicity tests of Kurlovs et al. (2022) previously revealed that the parental strain ROS-ITi was more than 90-fold resistant to dicofol, compared to the parental strain JP-RRi (Table 1)^27^. Females of the susceptible parental strain JP-RRi were crossed with males of the resistant parental strain ROS-ITi. The F_1_ progeny was expanded and the LC_50_ of dicofol for this segregating population was 465 mg a.i. L^-1^ (Table 1). This population was subsequently used to set up ten subpopulations without selection (control) and ten paired populations under dicofol selection. After 5 months of selection, phenotyping with a toxicity bioassay and a discriminating dose of 1000 mg a.i L^−1^ dicofol, revealed that unselected populations had a significantly higher mortality rate compared to their control populations (F_1,74_ = 78472, *p* < 2.2 e^−16^, Fig. 2a).

**Table 1:** Dicofol, bromopropylate and chlorobenzilate LC_50_ values for the inbred strains ROS-ITi, JP-RRi, and the segregating cross JP-RRi x ROS-ITi.

Compound	Strain	n	χ² (df)	Slope (±SE)	LC_50_^a^(95% CI)	RR (95% CI)
Dicofol	ROS-ITi^b^	990	33.192 (38)	1.575 (±0.196)	3125.380 (2398.290 – 4452.645)	92.304 (66.032 – 129.029)
	JP-RRi x ROS-ITi	408	18.520 (17)	3.460 (±0.324)	465.288 (404.964 – 532.003)	13.43 (11.28 – 15.99)
	JP-RRi^b^	517	24.754 (18)	4.050 (±0.487)	33.860 (27.487 – 39.838)	—
Bromopropylate	ROS-ITi	502	14.49 (18)	1.018 (±0.21)	1183.49 (653.31 – 4321.80)	21.25 (9.28 – 48.65)
	JP-RRi	402	36.89 (18)	3.407 (±0.38)	55.70 (45.19 – 68.55)	—
Chlorobenzilate	ROS-ITi	552	32.00 (18)	2.592 (±0.27)	249.58 (204.74 – 306.96)	2.14 (1.77 – 2.59)
	JP-RRi	460	39.21 (18)	3.669 (±0.47)	116.58 (88.84 – 140.15)	—

*n* number of mites, *SE* standard error, *CI* confidence interval, *RR* resistance ratio relative to JP-RRi, *df* degrees of freedom.

^a^mg a.i. L^-1^.

^b^toxicity data of dicofol (35% Kelthane) was retrieved from Kurlovs et al.^27^.

#### Genomic responses to dicofol selection

Genomic DNA was extracted from each of the selected and unselected populations and 24-42 million paired-end genomic reads were generated for each sample, while 45 and 29 million reads had previously been generated for the parental strains ROS-ITi and JP-RRi respectively^27^. Variants were called and merged across all samples in order to obtain an experiment-wide matrix containing allele frequencies of 570,828 segregating SNP and small indels. A PCA based on this matrix showed that 68.4% of the total variation could be explained by principal component 1 (PC1), while 3.0% could be explained by PC2 (Fig. 2b). Replicates grouped by selection procedure (dicofol selected vs unselected controls) on PC1 that explains the majority of variation in the dataset.

To investigate genomic regions that respond to dicofol selection, differences in allele frequencies between selected and control populations were assessed as outlined in^28^. Genome-wide allele frequencies between dicofol selected and unselected (control) populations revealed large deviations in allele frequencies on chromosome 2. Two significant (FDR < 0.05) sharp BSA peaks could be distinguished: at ~2.862 Mb (QTL1) and at ~19.907 Mb (QTL2) (Fig. 2c) (raw output is provided as a supplementary file on FigShare 10.6084/m9.figshare.23668188)^29^. Noteworthy, the ROS-ITi haplotype almost reached fixation at both QTL peaks (Supplementary Fig. 1) and was almost completely lost in the control samples at QTL1.

*TuGluCl3* (*tetur10g03090*) was located under the averaged BSA peak for QTL1 (Supplementary Data 1). This gene encodes a subunit of the GluCl receptor, known as the target-site of macrocyclic lactone acaricides such as abamectin and milbemectin^30–34^. Analysis of the allele frequencies of variants in *TuGluCl3* revealed that a previously documented abamectin resistance mutation (G326E) is present—and fixed—in ROS-ITi and the dicofol selected replicates, and absent in JP-RRi and the unselected control replicates^30,32^ (Supplementary Data 1, Supplementary Data 2). *TuGluCl1* (*tetur02g04080*), another GluCl gene, was found at a distance of 337 kb of the averaged BSA peak for QTL2 (Supplementary Data 3). As for *TuGluCl3*, a previously reported abamectin resistance mutation (G314D) in *TuGluCl1* was enriched in the selected populations, present in ROS-ITi and absent in JP-RRi^30,32,35^. Of particular note, both QTL1 and QTL2 of our BSA experiment showed nearly perfect overlap with two out of four QTLs that have recently been associated with abamectin resistance^32^ (Fig. 2c). In addition, two independent BSA experiments were performed in the abamectin study and both QTLs of this study were identified in each BSA experiment of Villacis-Perez et al. (2023)^32^. Finally, in addition to GluCl genes, other candidate resistance genes could be found in close proximity of the averaged BSA peak for QTL2, including genes encoding a UDP-glycosyltransferase (*tetur02g0330*), a PLAT/LH2 single domain protein (*tetur02g03490*), G-protein coupled neuropeptide receptors (GnRH-R4, *tetur02g03910* and NP-R8, *tetur02g03830*), a protein with a tumor necrosis factor receptor-associated factor (TRAF)-like domain (InterPro domain IPR008974; *tetur02g03390*), two Ser/Thr kinase TGFB receptors (TeturTGFbR1, *tetur02g03540*, and TeturTGFbR2, *tetur02g03540*) and a dual specificity phosphatase 14 (*tetur02g03440*). Lastly, *tetur02g03420* was located directly below the averaged BSA peak for QTL2 and its encoding protein showed a best BLASTp hit (E-value of 0 and 1e-43) with dumpy-PX (dpy-PX) and FBN-1, isoform g (a homolog of human Fibrilin-1) of *D. melanogaster* and *C. elegans* in FlyBase^36^ and WormBase^37^, respectively. To our knowledge, *D. melanogaster dumpy*-mutants have not yet been associated with avermectin resistance but, *C. elegans* Dumpy (Dpy) phenotypes, on the other hand, showed enhanced susceptibility to ivermectin^38^.

### TuGluCl3 and DmGluClα electrophysiology assays

GluCl genes, *TuGluCl3* (*tetur10g03090*) and *TuGluCl1* (*tetur02g04080*), were identified as potential resistance genes in QTL1 and QTL2, suggesting that dicofol acts on the GluCl receptor. To gain more insight into the effect of dicofol on the GluCl receptor, homomeric WT and G326E TuGluCl3 receptors were functionally expressed in *Xenopus* oocytes followed by two-electrode voltage clamp electrophysiology (TEVC). Only TuGluCl3 was investigated in this study, since our previous research failed to express an active TuGluCl1 receptor^31^. Additionally, a WT and G326E Drosophila GluCl receptor, DmGluClα, was included as a reference.

**Fig. 3:**
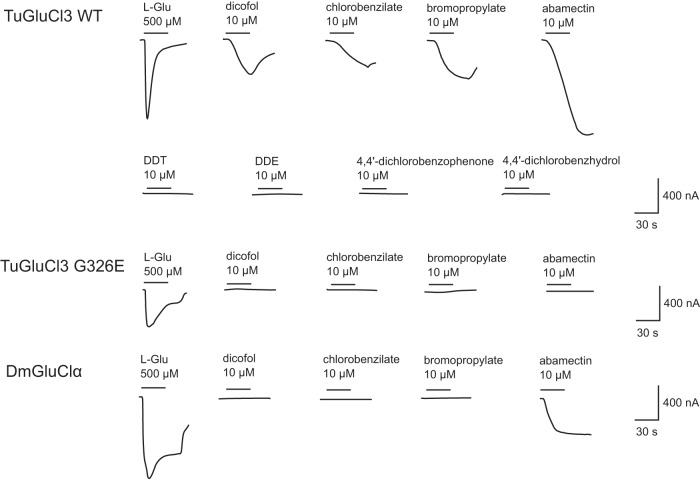
Examples of electrical current responses resulting from activation of TuGluCl3 WT and TuGluCl3 G326E receptors expressed in *X. laevis* oocytes by the natural agonist L-glutamic acid and various compounds. The period of compound application time is indicated by the bar above the trace as well as the concentrations applied (µM).

First, the response of these receptors to 500 µM of the natural agonist L-Glu and 10 µM of either diphenylcarbinol acaricides (dicofol, chlorobenzilate or bromopropylate), the avermectin acaricide abamectin, the voltage-gated sodium channel modulators DDT and metabolite DDE, or dicofol metabolites 4,4′ dichlorobenzophenone and 4,4′ dichlorobenzhydrol, was tested^39^ (Fig. 3). As previously observed in Mermans et al. (2017), both TuGluCl3 receptors showed clear responses to L-Glu^31^. The application of abamectin and the diphenylcarbinol acaricides dicofol, chlorobenzilate and bromopropylate elicited clear inwards currents in the TuGluCl3 WT receptor. No response was recorded when exposing the TuGluCl3 WT receptor to DDT, DDE, 4,4’-dichlorobenzophenone or 4,4′-dichlorobenzhydrol. In contrast, the TuGluCl3 G326E receptor was not activated by any of the acaricides or metabolites tested, even when tested at high concentrations (10 µM). Further, the responsiveness of both DmGluClα WT and G326E receptors was tested, with DmGluClα WT only showing clear responses to L-Glu and abamectin. All other tested compounds, including dicofol, could not activate the DmGluClα WT receptor. The DmGluClα G326E receptor failed to generate any response, even to its natural agonist L-Glu, potentially indicating that we could not functionally express this receptor successfully. The latter is in line with Xue et al. (2020), where the introduction of G326E into GluClα of *Drosophila* flies was lethal^34^.

**Fig. 4:**
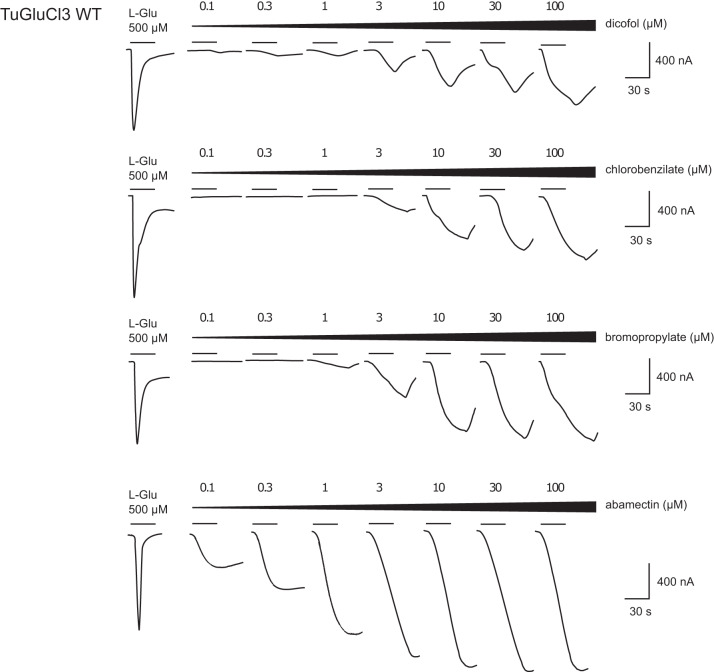
Agonistic activity of dicofol, chlorobenzilate and bromopropylate on the TuGluCl3 WT receptor expressed in *X. laevis* oocytes. The curves show the current traces after exposure to increasing dosages of dicofol, chlorobenzilate, bromopropylate or abamectin. The bars indicate the time period of application of L-glutamic acid and increasing concentrations of the three tested substances (100 nM–100 μM).

**Fig. 5:**
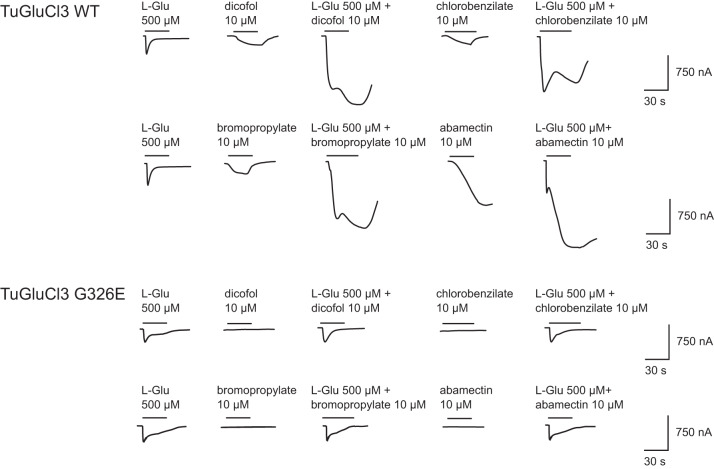
Dicofol, bromopropylate, chlorobenzilate and abamectin potentiation of currents induced by L-glutamic acid in TuGluCl3 WT and TuGluCl3 G326E receptors. Oocytes expressing the TuGluCl3 WT or TuGluCl3 G326E receptor were perfused with 500 µM of L-glutamic acid followed by co-application of 500 µM L-glutamic acid and 10 µM of acaricide dicofol, bromopropylate, chlorobenzilate or abamectin.

Finally, to investigate the response of TuGluCl3 WT receptor to dicofol, chlorobenzilate and bromopropylate in detail, averaged dose-response curves were obtained for the three compounds (Supplementary Fig. 2) and EC_50_s are listed in Table 2 (Fig. 4). The statistical analysis indicated that there was no significant difference in the EC_50_s of dicofol, chlorobenzilate, and bromopropylate. However, these values differed when compared to the response of abamectin and L-Glu (Supplementary Data 4). Dicofol, chlorobenzilate, bromopropylate and abamectin were also co-applied with L-Glu to examine possible potentiation of glutamate-induced currents. A clear potentiation of the currents for all compounds was demonstrated for TuGluCl3 WT, while none of the compounds could potentiate glutamate response in homomeric TuGluCl3 G326E receptors (Fig. 5). Potentiation ratios for TuGluCl3 WT are listed in Supplementary Data 5.

**Table 2:** EC_50_ values for dicofol, chlorobenzilate, bromopropylate and abamectin tested on TuGluCl3 WT receptors expressed in *X. laevis* oocytes.

Compound	n	EC_50_ (µM)	pEC_50_	^a^nH
Dicofol	6	3.583	5.446 ± 0.087	1.3 ± 0.33
Chlorobenzilate	6	2.779	5.556 ± 0.040	2.2 ± 0.48
Bromopropylate	6	3.231	5.491 ± 0.059	1.8 ± 0.47
Abamectin^b^	8	0.447	6.32 ± 0.12	1.0 ± 0.23

^a^n_H_: Hill coefficient.

^b^Data previously published in^34^.

### Cross-resistance evaluation

Considering the similar response of the TuGluCl3 WT receptor to dicofol as well as to bromopropylate and chlorobenzilate, the toxicity of the latter two compounds towards *T. urticae* was evaluated in toxicity bioassays on the BSA parental strains ROS-ITi and JP-RRi (Table 1). Resistance levels between strains ranged from 20-fold for bromopropylate to 2-fold chlorobenzilate, confirming cross-resistance between all 3 compounds.

### Homology modeling and molecular docking

#### Docking analysis of the compounds with TuGluCl3 WT and G326E

Given that abamectin is known to act on the GluCl receptor (Fig. 2) and that a G326E mutation in  the GluCl receptor abolishes the agonistic activity of abamectin^31–33,40^, we first evaluated homology modeling and molecular docking experiments with TuGluCl3 and abamectin. The two components of abamectin; avermectin B1a (AVM B1a) and B1b (AVM B1b), were docked into TuGluCl3 WT and G326E (Table 3). As shown in Supplementary Figure 3, the binding conformation of AVM B1a was similar to AVM B1b, with the binding pocket located between TM3 of chain A (TM3/A) and TM1 of chain B (TM1/B). The hydroxyl group of AVM B1a formed a strong hydrogen bond with the carbonyl group of A302 (bond length: 1.9 Å) and the oxygen atom on the hydroxyl group of S306 (bond length: 2.0 Å) on TM3/A, respectively. In addition, there was a π-π stacking interaction (distance: 4.9 Å) between the furan ring of AVM B1a and the phenyl ring of F333. Interestingly, the docking scores of the two components with TuGluCl3 G326E were considerably lower than those with TuGluCl3 WT, indicating that the binding affinity of both AVM compounds was significantly weakened after G326 was mutated into E326 (Table 3). AVM B1a and AVM B1b were both outside the binding pocket of TuGluCl3 G326E and formed no interaction with the active site (Supplementary Fig. 3). As the mutation from G326 to E326 resulted in a volume change of the side chain, leading to a large steric hindrance, the entrance of the active site was blocked and the compound failed to extend into the binding pocket to form interactions.

**Table 3:** The docking scores and binding free energies of dicofol, bromopropylate, chlorobenzilate, DDT and abamectin components AVM B1a and AVM B1b with TuGluCl3 wild type (WT), TuGluCl3 G326E and DmGluClα.

Compound	Docking score			Binding energy (kJ mol^-1^)		
	TuGluCl3		DmGluClα	TuGluCl3		DmGluClα
	WT	G326E		WT	G326E	
Dicofol	6.85	3.86	3.49	−39.09	−22.02	−19.91
Bromopropylate	5.09	3.03	—^a^	−29.04	−17.29	—
Chlorobenzilate	5.19	2.95	—	−29.61	−16.83	—
DDT	2.71	—	—	−15.46	—	—
AVM B_1a_	6.65	2.48	6.42	−37.94	−14.15	−36.63
AVM B_1b_	6.63	2.36	6.37	−37.83	−13.47	−36.35

^a^a horizontal line indicates not assessed.

**Fig. 6:**
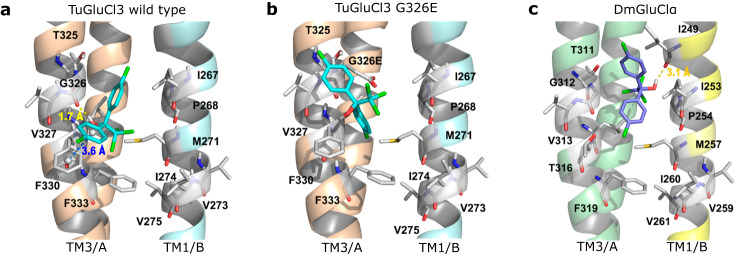
The binding pattern of dicofol in the active site of GluCl receptors. **a** Binding pattern of dicofol in the active site of TuGluCl3 WT. **b** Binding pattern of dicofol in the active site of TuGluCl3 G326E. **c** Binding pattern of dicofol in the active site of DmGluClα. The protein is shown as a cartoon. The hydrogen bond and π-π stacking are shown as yellow and blue dashes, respectively.

Next, homology modeling and molecular docking was used to determine the impact of the G326E mutation on the binding of TuGluCl3 with dicofol and chemically related compounds—bromopropylate and chlorobenzilate. According to the docking results, dicofol showed the highest docking score and the lowest binding free energy—followed by chlorobenzilate and bromopropylate—and dicofol had a similar binding affinity as AVM B1a and AVM B1b with TuGluCl3 WT (Table 3). All compounds were embedded in an active cavity between TM3 of Chain A (TM3/A) and TM1 of Chain B (TM1/B) (Fig. 6 and Supplementary Fig. 4). The hydroxyl group of dicofol formed a strong hydrogen bond with G326 of wild-type TuGluCl3 (bond length: 1.7 Å), and there was a strong π-π stacking interaction (distance: 3.6 Å) between its benzene ring and F330. The hydroxyl groups of bromopropylate and chlorobenzilate also formed a strong hydrogen bond with the carbonyl group of G326. Once G326 was mutated into E326, the α-hydrogen atom of glycine was replaced by a carboxyethyl group, which enlarged the volume of the side chain and consequently caused steric hindrance in the active site. Due to the steric hindrance, the chlorophenyl group of dicofol failed to probe into the cavity near E326, and the overall conformation of the compound was inverted, resulting in the larger space between the hydroxyl group and E326, preventing the formation of the hydrogen bond dicofol and E326. Moreover, the π-π stacking interaction between dicofol and F330 also disappeared after the mutation. Likewise, the mutation prevented the chlorophenyl groups of bromopropylate and chlorobenzilate from penetrating into the active site.

As the only structural difference between dicofol and DDT is the presence of a hydroxyl group in the latter, also the binding of DDT with TuGluCl3 was studied. According to the docking results, DDT and dicofol were both inserted into the active site between TM2/A, TM3/A, and TM1/B, but with very different binding modes, despite their subtle structural difference. Although DDT was in the same binding pocket as dicofol, no hydrogen bond was formed to stabilize its binding to the receptor due to the lack of hydrogen bond donors at the linker between the two benzene rings, resulting in a more flexible conformation of DDT and one of its chlorobenzene rings extended deeper into the active cavity (Supplementary Fig. 5).

#### Selectivity analysis of dicofol and abamectin to TuGluCl3 and DmGluClα

Homology modeling showed that the secondary structures of the TuGluCl3 and DmGluClα receptors are highly similar. Dicofol binding modes also seemed similar as key residues around the binding pocket of dicofol in TuGluCl3—T325, G326, V327, and F330 of TM3/A, and I267, P268, and M271 of TM1/B—corresponded to those in DmGluClα—T311, G312, V313, and T316 of TM3/A, and I253, P254, and M257 of TM1/B. However, according to the docking results, dicofol in TuGluCl3 showed a higher docking score and lower binding free energy than in DmGluClα, indicating that dicofol might have stronger binding affinity with TuGluCl3 (Fig. 6). Indeed, in TuGluCl3 dicofol formed a strong hydrogen bond with G326 and a strong π-π stacking interaction with F330 (Table 3), whereas the hydroxyl group of dicofol in DmGluClα was far away from the carbonyl group of G312, so there was no hydrogen bond interaction between dicofol and the binding pocket of DmGluClα. Moreover, since the residue type at residue 316 of DmGluClα (corresponding to residue 330 in TuGluCl3) was different (threonine instead of phenyl-alanine), there was no π -π stacking between dicofol and DmGluClα. In addition, a hydrogen bond (bond length: 3.1 Å) was formed between the hydroxyl group of dicofol and I249 on TM1/B of DmGluClα, which is a less stable bond with longer distance compared to the hydrogen bond between dicofol and G326 of TuGluCl3.

AVM B1a and AVM B1b were both embedded in the binding pocket between TM3/A and TM1/B of DmGluClα, similar to TuGluCl3 (Supplementary Fig. 3). The disaccharide groups of AVM B1a and AVM B1b were completely inserted into the binding pocket and other parts were partially outside the pocket to form hydrophobic interactions. A hydrogen bond was formed between the hydroxyl group of AVM B1a and A288 (bond length: 2.1 Å) and between AVM B1a and S292 (bond length: 2.0 Å of DmGluClα. In addition, a π-π stacking interaction (distance: 4.3 Å) was formed between the furan ring of AVM B1a and the phenyl ring of F319. Similarly, strong hydrogen bonds were also formed between AVM B1b and the residues A288 (bond length: 2.2 Å) and S292 (bond length: 2.3 Å) as well as a π-π stacking interaction between its furan ring and the phenyl ring of F319 (distance: 4.9 Å) of DmGluClα. These docking results indicated that, in contrast to dicofol, AVM B1a and AVM B1b had a good binding affinity with both TuGluCl3 and DmGluClα.

## Discussion

A previous resistance screening study revealed that a European *T. urticae* strain, ROS-ITi, was highly resistant against dicofol^27^. Such high resistance levels were unexpected, as dicofol has been banned for agricultural use in Europe since 2008^41^. The identification of cross-resistance mechanisms that might be at play could be relevant for resistance management in other regions in the world where dicofol is still being used. To investigate dicofol resistance mechanisms in this strain without any prior hypothesis, we performed a high-resolution genetic mapping experiment. For this purpose, we crossed the resistant ROS-ITi strain to an inbred susceptible strain, and subjected the segregating population to increasing concentrations of dicofol in an experimental evolutionary setup (evolve and resequence). Two genomic regions on chromosome 2—QTL1 at ~2.86 Mb and QTL2 at ~19.9 Mb—could be associated with dicofol resistance. Surprisingly, these two QTLs showed a clear overlap with two QTLs uncovered in two independent experiments mapping abamectin resistance by Villacis-Perez et al. (2023) (Fig. 2), and points towards similar resistance mechanisms for both dicofol and abamectin. Cross-resistance between these compounds was indeed observed for ROS-ITi^27^. In addition, the overlapping QTLs of Villacis-Perez et al. (2023) and this study each harbor a GluCl gene—*TuGluCl3* (*tetur10g03090*) at QTL1 and *TuGluCl1* (*tetur02g04080*) at QTL2—and both genes contain previously documented abamectin resistance mutations^30–32,35^. Together, these data suggest that dicofol and abamectin might share a target-site and that mutations that confer resistance to abamectin, also confer cross-resistance to dicofol.

To further reinforce the likelihood that the GluCl receptor is the target-site of dicofol, we expressed the *T. urticae* GluCl3 receptor in *Xenopus* oocytes and performed extensive electrophysiological experiments. TEVC assays showed that dicofol and other diphenylcarbinol acaricides, such as chlorobenzilate and bromopropylate, elicit clear inwards currents in the TuGluCl3 WT receptor. These responses were not observed with DDT and the other metabolites tested, but were observed with abamectin, clearly suggesting both diphenylcarbinol acaricides and abamectin interact with the GluCl receptor. Moreover, the TuGluCl3 G326E mutant receptor was not activated by any of the allosteric agonists even when tested at high concentrations, indicating that this mutation drastically alters the interaction with abamectin, dicofol, chlorobenzilate and bromopropylate. Although we did not manage to express *TuGluCl1*, located at QTL2, similar patterns of activation and loss of interaction due to a G314D resistance mutation most likely occur. Further, given that dicofol is a selective acaricide, mainly working on mites with limited to no activity on insects^1,42^, it seemed interesting to test whether this specificity is target-site specific. Therefore, we also expressed the *Drosophila melanogaster* GluCl receptor (DmGluClα) and performed TEVC assays. As expected, strong inward currents were observed for abamectin, but these were not observed for any other tested acaricide, suggesting that the specificity of this class of compounds is due to target-site specificity of GluCls.

The QTL mapping experiment and TEVC assays clearly point towards the GluCl receptor as a mite-specific shared target-site of the diphenylcarbinol acaricides dicofol, chlorobenzilate, bromopropylate and the structurally unrelated acaricide abamectin. To complement these findings and obtain insights in the mechanisms of selectivity, we studied the interaction of these acaricides with the GluCl receptor via homology modeling of the *T. urticae* and *Drosophila* GluCl receptor, combined with ligand-docking. This revealed a common binding pocket between WT TuGluCl3 TM3 of chain A (TM3/A) and TM1 of chain B (TM1/B) of all studied compounds, although with different binding affinities. The results for abamectin are consistent with the knowledge that avermectins act on TM3 and TM1 of two adjacent GluCl subunits except that in our study the docking position of the disaccharide moiety is opposite; in the crystal structure, the disaccharide moiety is docked outside the binding site, whereas in the homology model, this moiety appears to be inserted into the binding site^43^. However, for the TuGluCl3 G326E mutant receptor, docking scores of all compounds were drastically reduced as glutamic acid has a larger volume, causing steric hindrance in the active site. For dicofol, the G326E mutation resulted in the loss of a key hydrogen bond and π-π stacking interaction that helped stabilize the binding with TuGluCl3. Moreover, due to the conformational changes of the other diphenylcarbinol acaricides, the hydroxyl groups of both bromopropylate and chlorobenzilate failed to form any hydrogen bond with E326. For abamectin compounds AVM B1a and AVM B1b, the conformations of the two components were greatly deflected in binding to TuGluCl3 G326E, but neither of them could break through the blockage of the active site successfully. As an alternative to changes in ligand binding affinities, the G326E mutation could also affect the intrinsic—channel gating—properties of the receptor with no specific effect on dicofol or AVM B1 binding, as was previously shown for another macrocyclic lactone (ivermectin) and a glycine to alanine substitution at the corresponding residue in the GluCl receptor of *Haemonchus contortus* (avr-14b)^44^. However, in our study, glycine is not replaced with a small amino acid (alanine) but with a large amino acid (glutamic acid), suggesting that steric hindrance is probably the major effect of the substitution, as modeling suggests, although effects on channel gating cannot be ruled out.

Compared to TuGluCl3, the binding affinity of dicofol in DmGluClα was drastically reduced due to an unstable hydrogen bond and the absence of π-π stacking, whereas the affinity of abamectin components remained equally strong in DmGluClα. Interestingly, this absence of π-π stacking in DmGluClα was due to an amino acid change at residue 316 (threonine instead of phenyl-alanine; compared to TuGluCl3). In silico screening revealed that at least one GluCl gene of (true and false) spider mites does have a phenyl-alanine at the corresponding residue, but other GluCl genes of spider mites and all GluCl genes of eriophyoid mites and *V. destructor*, for which dicofol is also an effective acaricide^45,46^, do have a different amino acid at this position (Supplementary Fig. 6). This suggests that other interactions than those with phenyl-alanine might also be important for dicofol selectivity. To conclude, our modeling results shed light on why the G326E mutation in TuGluCl3 confers resistance to abamectin, dicofol, bromopropylate and chlorobenzilate, but also confirms the selectivity of dicofol towards mite-specific GluCl receptor.

Our results show that compounds that are very similar can display a completely different mode of action, and one should be cautious when assuming the same target-site for structurally related compounds^47^. DDT and dicofol only differ by a hydroxyl group that replaces a hydrogen at the carbon atom located between the two benzene rings of DDT (Fig. 1). We showed that DDT failed to form interactions with the surrounding residues at the TuGluCl3 binding site and its binding was less stable than dicofol for which the hydrogen bond between the hydroxyl group and G326 contributed to the excellent activity. Many historical studies, with various insect species, have shown that enzymatic metabolization of DDT to dicofol via oxidative hydroxylation plays a part in DDT resistance^48–53^. Indeed, this different molecular shape and size has shown to cause a loss in affinity to the sodium channel and in toxicity in houseflies^54^. Also Seiber & Kleinschmidt (2010) already emphasized, with the comparison between DDT and dicofol, that a small structural change in the chemical structure can have major effects on the biological activity and the environmental fate of a product^55^. That a small difference in molecular structure can cause a great shift in response of *T. urticae* GluCl3 is also clear from comparing the response between chlorobenzilate and 4,4′-dichlorobenzohydrol in the TEVC assays, as the lack of an ethoxycarbonyl group of chlorobenzilate to form 4,4′-dichlorobenzohydrol results in the loss of GluCl3 agonism. In line with this observation, docking results suggest that the lack of the ethoxycarbonyl group creates a space in the binding site, probably making it more difficult to interact with nearby amino acid residues, changing the docking pose and resulting in the binding destabilization of 4,4′-dichlorobenzhydrol in the cavity. However, care must be taken when interpreting docking results, as these only provide a “snapshot” and the actual binding is probably more dynamic.

The discovery of GluCls as a common target-site for abamectin, dicofol and other diphenylcarbinol acaricides and the fact that similar resistance mutations cause cross-resistance, is also supported by toxicity data. First, the ROS-ITi strain was highly resistant to both abamectin and dicofol but also showed resistance to bromopropylate and chlorobenzilate. Many previous studies documented cross resistance between dicofol, bromopropylate and chlorobenzilate, that in hindsight might be due to target-site resistance^10–12,56–59^. High synergism between the diphenylcarbinol bromopropylate and avermectins was also recorded in the citrus red mite^60^. At the time of commercialization of abamectin, it was also suggested that common resistance mechanisms for dicofol and abamectin resistance might evolve in the field^61^. It is tempting to hypothesize, but hard to prove, that the known abamectin resistance mutations might actually have been first selected by dicofol and not by avermectins.

Last, similar to dicofol, bromopropylate and chlorobenzilate were reported to be very selective as both acaricides were found toxic to two-spotted spider mites whereas insects, such as *Musca domestica*, were not affected by these compounds^1,42^. Also Jeppson (1955) emphasized the selectivity of chlorobenzilate as it has a low toxicity to warm-blooded animals, insect parasites and predators or bees while it effectively controls citrus mites^62^. The selectivity of the diphenylcarbinol acaricide group is now better understood given their common selective mode of action via GluCls.

GluCl mutations as resistance mechanisms might also allow to better understand some historical data on the stability of dicofol resistance^20,63,64^. Dennehy et al. (1990) reported that dicofol is still very effective in the control of spider mites even after 25 years of usage and, together with Inoue (1979), they observed a much lower relative fitness of the dicofol resistant population than the dicofol susceptible population^65,66^. This is in line with Bajda et al. (2018) investigating fitness of abamectin resistance mutations in GluCls. By backcrossing these mutations into a susceptible genomic background, this study revealed a significant negative impact on population growth associated with the target-site mutations G314D and G326E in TuGluCl1 and TuGluCl3 respectively^67^. Noteworthy, also in this study we found that the haplotype of the resistant parent reached complete fixation at both QTL peaks (Supplementary Figure 1; Supplementary Data 2) and was almost completely lost in the control samples at QTL1, hinting towards a potential fitness cost of the selected loci without continuous selection pressure. The presence of a fitness cost of target-site resistance mutation would explain the sustained efficacy of dicofol and abamectin, despite the first report of dicofol resistance already dates from 1965 and the first abamectin resistance case has only been reported in 1998^19^. Of course, our findings do not rule out other mechanisms for dicofol resistance in unrelated strains as historically dicofol resistance has mainly been linked with increased metabolic detoxification^10,24,25^. Moreover, contrary to the two genomic loci shown to underlie dicofol resistance in *T. urticae*, several crossing experiments assessing the inheritance mode of dicofol resistance in spider mites^68–71^, propose a monogenetic inheritance of dicofol resistance.

The correlation between the presence of GluCl mutations in GluCl genes and abamectin resistance is very clear from multiple studies^30–32,34,35,40,72–74^. However, it has also been documented that the combined presence of *TuGluCl1* and *TuGluCl3* mutations does not lead to high levels of abamectin resistance in vivo, despite convincing TEVC data for the G326E substitution in homomeric TuGluCl3 receptors^31,73^. Xue et al. (2020) postulated that the unknown complexity of hetero-pentameric GluCl receptors, possibly consisting of non-GluCl subunits^75^, might be the main reason for the limited phenotypic strength of GluCl mutations with regard to abamectin resistance. Moreover, multiple GluCl genes might have tissue specific expression giving rise to varying phenotypic strength. In addition, it was clear that high abamectin resistance levels could only be attained in the presence of an additional resistance mechanism, most likely increased detoxification. This is supported by the detection of 4 QTLs in Villacis-Perez et al.^32^, and a cytochrome P450 mono-oxygenase (CYP392A16) that is frequently overexpressed in abamectin resistant strains and can metabolize abamectin to a non-toxic compound^76,77^. Although genetic mapping pointed towards a role of both GluCl mutations in dicofol resistance of ROS-ITi, it would also be interesting to assess the relative contribution of each GluCl mutation, and their combination, to dicofol resistance. In addition, as *TuGluCl1* was located far (about 300 kb) away from the averaged BSA peak of QTL2, one can also not exclude that other genes than those encoding the target-site might play role in dicofol resistance of ROS-ITi.

To conclude, using an integrative approach—including QTL mapping experiments, TEVC assays and 3D modeling and docking—we showed that the GluCl receptor is the mite specific target-site of dicofol and other diphenylcarbinol acaricides. A substitution in the GluCl receptor abolishes activity of diphenylcarbinol acaricides and abamectin which provides evidence for a common target-site. Considering the historical use of dicofol ( > 20 years before abamectin), dicofol resistance mutations could have resulted in rapid development of abamectin resistance. This was probably counteracted by fitness costs that are associated with GluCl resistance mutations and the fact that at least for abamectin, high resistance ratios can only be attained in synergy between target-site resistance and increased detoxification.

## Materials & methods

### Acaricides and chemicals

The acaricide used in this study was a commercial formulation of dicofol (Kelthane, 35% WP). All other chemicals, including the L-glutamic acid monosodium salt monohydrate (CAS number 6106-04-3) and the analytical standards of dicofol (CAS number 115-32-2), abamectin (CAS number 71751-41-2, more than 80% avermectin B1a and less than 20% avermectin B1b), DDT (CAS number 50-29-3), DDE (CAS number 72-55-9), chlorobenzilate (CAS number 510-15-6), bromopropylate (CAS number 18181-80-1) were purchased from Sigma–Aldrich (Belgium). Previously reported metabolites of dicofol, including 4,4′-dichlorobenzophenone (CAS number 9990-98-2) and 4,4′-dichlorobenzhydrol (CAS number 90-97-1)^25,78,79^ were also purchased from Sigma–Aldrich (Belgium).

### Mite strains and husbandry

The *T. urticae* inbred strains used in this study were green color morph strains and were previously described in Kurlovs et al. (2022)^27^. The ROS-ITi strain was highly resistant to dicofol while the JP-RRi strain was highly susceptible to dicofol. All mite strains were maintained on potted kidney bean plants (*Phaseolus vulgaris* L. cv. Prelude) in a climatically controlled room or incubator at 25 ( ± 0.5) °C, 60% relative humidity, and 16:8 h light:dark photoperiod. During BSA experiments, experimental populations originating from the JP-RRi x ROS-ITi cross (see section 2.4 Bulked segregant analysis) were maintained on potted kidney bean plants placed in mite-proof cages (BugDorm-4F4590DH, MegaView Science Co., Taiwan) in a greenhouse at 20–25 °C and 50–80% relative humidity.

### Toxicity bioassays

Toxicity assays with dicofol and the expanded progeny of the initial BSA cross (JP-RRi x ROS-ITi, see below), and with bromopropylate or chlorobenzilate were performed as in Khajehali et al. (2011)^80^. Briefly, 20-30 adult female mites were placed on 9 cm^2^ square bean leaf disks placed on wet cotton. Technical standards of the acaricides bromopropylate and chlorobenzilate were dissolved in a mixture of N,N-dimethylformamide and emulsifier W (alkarylpolyglycolether), 3:1 w/w, respectively, and subsequently diluted 100-fold with deionized water. For LC_50_ determination, at least five different acaricide concentrations were tested in four-fold replication with demineralized water as control for dicofol, and blank formulation (1/100 dilution of mix of N,N-dimethylformamide and emulsifier W) as a control for bromopropylate and chlorobenzilate. Using a Cornelis spray tower, 800 µL fluid was sprayed on the mites on each leaf-disk at a pressure of 1 bar (which results in 1.5 ± 0.06 mg fluid deposition per cm^2^)^81^. The leaf disks were then placed in a climatically controlled room at 25 °C, 60% relative humidity and 16:8 h light:dark photoperiod. Mortality was assessed after 24 h. Mites were considered dead when they failed to respond to prodding with a camel’s hair brush. Concentration-response curves, LC_50_-values, slopes, 95% confidence intervals (CIs) and resistance ratios were calculated by probit analysis (POLOplus, LeOra Software, USA).

### Bulked segregant analysis

#### Experimental evolution setup

The initial cross of the BSA experiment was performed in Villacis-Perez et al. (2023)^32^, but while abamectin was used for selection in Villacis-Perez et al. (2023), BSA populations were selected with dicofol in this study. Briefly, 41 virgin females (teleiochrysalid stage) of the susceptible strain JP-RRi were placed together with 21 adult males of the resistant strain ROS-ITi on a detached bean leaf. The resulting F_1_ population was expanded on detached bean leaves for two generations until they were placed on potted bean plants in mite-proof cages (BugDorm-4F4590DH, MegaView Science Co., Taiwan) and allowed to grow under greenhouse conditions (20–25 °C and 50–80% relative humidity). After reaching sufficiently high population levels—two to three generations later—the LC_50_ of dicofol was assessed for the segregating population using the toxicity bioassay described in section Toxicity Bioassays. After the sixth generation, the population was split into 10 subpopulations of each 350 fertilized female mites and allowed to expand for two generations. Next, for each of the 10 subpopulations, 10 sister populations were set up in a paired design. This resulted in 10 populations that were selected with increasing concentrations of dicofol and 10 shared control populations that were allowed to grow without selection pressure (20 populations in total). The initial concentration for selection was 120 mg a.i. L^-1^ dicofol, while the final concentration for selection at the end of the experiment was 1000 mg a.i. L^-1^. Selection was performed by spraying uninfested bean plants with a hand-held spraying device (Birchmeier, Switzerland) until runoff and transferring mites to newly sprayed plants. After 14 generations dicofol toxicity was assessed for each subpopulation, control and selected, in a toxicity bioassay as described in section Toxicity Bioassays and using a discriminating dose of 1000 mg a.i. L^-1^ dicofol. Differences in survival rate between selected and control replicates were analyzed in R (version 4.0.4)^82^ using a linear mixed model (fixed factor = ‘treatment’, random factor = ‘replicate’) with a Satterthwaite’s approximation to calculate adjusted *p-*values. Simultaneously, 800 adult females were collected per population (14^th^ generation on plants with or without selection pressure). The total duration of the experimental evolution experiment was 5 months. Genomic DNA (gDNA) was isolated by chloroform-phenol extraction as previously described (Sambrook and Russell, 2001). Quality and quantity of the gDNA samples were assessed using a Denovix DS-11 spectrophotometer (DeNovix, USA) and by running a 2% agarose gel electrophoresis (30 min at 100 V).

#### DNA sequencing and bioinformatic analyses

Genomic sequence reads for both parental strains were previously generated and are publicly available in the NCBI Sequence Read Archive (SRA) under BioProject PRJNA799176^27^. For all the experimental BSA replicates of the dicofol selected and control populations, Illumina libraries were constructed using the TruSeq Nano DNA sample preparation kit and sequenced on an Illumina NovaSeq 6000 Sequencing platform, generating paired-end reads of 100 bp (perfomed at Fasteris, Switzerland). Genomic sequence reads of all dicofol-selected BSA replicates were deposited to the NCBI SRA under BioProject PRJNA990678. Genomic sequence reads for control BSA replicates were previously generated and are publicly available in the NCBI SRA under BioProject PRJNA930642^32^. For each sample, reads were aligned to the three pseudochromosome genome assembly of *T. urticae*^83,84^, using the default settings of the Burrows-Wheeler Aligner (BWA) (version 0.7.17-r1188)^85^ and processed into position-sorted BAM-files using SAMtools (version 1.11)^86^. Duplicates were marked using Picard tools (version 2.20.4-SNAPSHOT) (https://broadinstitute.github.io/picard). Joint variant calling across all 20 populations and the parental strains was done with GATK’s (version 4.1.4.1)^87^ HaplotypeCaller tool to produce a variant call format (VCF) file containing single nucleotide polymorphisms (SNPs) and indels. The VCF of each population/strain was subsequently merged into one VCF file using the GATK’s CombineGVCF tool and further processed to the final VCF file for BSA analyses using GATK’s GenotypeGVCF tool. The BSA analysis was performed using the “RUN_BSA1.02.py” script available at https://github.com/rmclarklab/BSA^28^ with default settings for paired offspring data, “-perm 10000”, “-sig 0.05” and the final VCF file as input. Genes within a 100 kb window of QTL1 were visualized using the R-package Gviz (version 1.40.1)^88^ with the *T. urticae* gff3 annotation of the gene models published by^84^. A principal component analysis (PCA) was performed in R (function ‘prcomp’ within the R package ‘stats’*;* version 2.3.0) as described in^89^. Briefly, a correlation matrix containing individual SNP frequencies for each population was used as input for ‘prcomp’. Only SNP’s that differentiated the two parental lines and that were present in all treatments (dicofol selected, control) were included in this correlation matrix. A two-dimensional PCA plot was created using the function ‘autoplot’ in R package ‘ggplot2’ (version 2.1.0)^90^.

### Potential effect of variant alleles in coding sequences

To predict the potential effect of genetic variants in loci identified in the BSA analysis, coding effects of SNPs and small indels—found in the GATK analysis—were predicted using SnpEff (version 5.0c)^91^ with a *T. urticae* database built using the three-pseudochromosome assembly reference genome^84^ and a coding sequence database derived from the June 23, 2016 annotation (available from the Online Resource for Community Annotation of Eukaryotes – ORCAE^92^). Within the SnpEff package, the SNPsift toolbox was used to filter the SNPeff output for variants present in the resistant parent line ROS-ITi, absent in the susceptible parent line JP-RRi and enriched in all selected populations.

### Two-electrode voltage clamp electrophysiology (TEVC)

#### Vector construction and cRNA synthesis

Wild type (WT) and TuGluCl3 G326E constructs for TEVC experiments are previously described in^31^ and synthesized by GenScript (USA). The same approach was used to generate a DmGluClα WT construct, using the *Drosophila melanogaster* GluClα coding sequence of Cully et al.^93^. DmGluClα G326E, containing a G - > E mutation (at residue 312 of DmGluClα) corresponding to the G326E mutation in TuGluCl3, was generated by Genscript using site-directed mutagenesis and the DmGluClα WT construct as template. All GluCl coding sequences were preceded with a KOZAK sequence (‘GCCAC’) and codon optimized for *Xenopus* expression using the OptimumGene™-Codon Optimization software of GenScript (sequences of codon optimized DmGluClα constructs are given in Supplementary Data 6). cRNA synthesis was carried out as previously described^31,34^. Quality and quantity of cRNA was evaluated via a Denovix DS-11 spectrophotometer (DeNovix, USA) and agarose gel electrophoresis and cRNA was stored at −80 °C until use.

#### Oocyte injection and two-electrode voltage clamp electrophysiology

Defolliculated, stage V–VI *Xenopus laevis* oocytes (Ecocyte Bioscience, Germany), in Tris-buffered Barth’s solution (Ecocyte Bioscience, Germany), were microinjected using a Nanoject III Programmable Nanoliter Injector (Drummond Scientific Co., USA) with 75 nL of cRNA solution (25—50 ng μL^−1^) and incubated at 18 °C in sterile Barth’s solution for a minimum of 24 h before experimentation, as previously described^34,94^. Optimal expression was achieved at 2–3 days post-cRNA injection. Two-electrode voltage-clamp (TEVC) recordings were made using the fully automated Roboocyte2 (Multi Channel Systems MCS GmbH, Germany). Oocytes were held in a standard 96-well microtitre plate and impaled with two glass microelectrodes filled with 0.1 M KCl 1.5 M potassium acetate solution to yield a resistance of ∼1 MΩ. Oocyte membrane potentials remained fixed at −60 mV throughout the experiment.

Test solutions of the natural agonist L-glutamic acid monosodium salt monohydrate (L-Glu) and the technical standards of dicofol, DDT, DDE 4,4′-diclorobenzophenone, 4,4′-dichlorobenzhydrol, chlorobenzilate, bromopropylate and abamectin were freshly prepared as 1 mM stock solutions made with dimethyl sulfoxide (DMSO) and diluted with Normal Frog Ringer (NFR) solution (Ecocyte Bioscience, USA) to a final concentration of 1% DMSO. Preliminary tests to study the channels’ responsiveness were performed by exposing the oocytes to 500 µM L-Glu followed by 10 µM of every compound for 30 s with a 60 s recorded wash-out (NFR) in between applications to allow the current to return to baseline. Next, dose-response curves for responsive agonists were obtained by sequential applications for 30 s of increasing concentrations followed by a 60 s recorded wash-out. All agonist applications were preceded by L-Glu (at EC_50_ concentration) to normalize the response and to validate GluCl expression. Next, dicofol, chlorobenzilate, bromopropylate and abamectin were co-applied with L-Glu for 30 s to injected oocytes to test agonist potentiation of glutamate-induced currents. Potentiation ratios were calculated by dividing the response (nA) of co-application by the response to L-Glu. Responses to L-Glu applications were normalized (I% = (I/Imax) × 100) and graphed as means ± SEM (standard error of the mean) using at minimum of 6 oocytes. TEVC recordings were analyzed using the Roboocyte 2+ software (version 1.4.3) (Multi Channel Systems MCS GmbH, Germany), EC_50_, pEC_50_ values and Hill coefficients were calculated by fitting a four-parameter logistic curve (Hill equation) on response data using SigmaPlot software (version 13.0) (Systat Software, USA) and GraphPad Prism software (Dotmatics, USA). Statistical comparison of EC_50_ values was done in R (version 4.2.2) using a Kruskal-Wallis analysis followed by pairwise comparisons using Wilcoxon rank sum exact test with Benjamini-Hochberg adjustment of *p*-values (Supplementary Data 4). A *p*-value < 0.05 was considered significant.

### Homology modeling and molecular ligand docking

The homology models of TuGluCl3 and DmGluClα were constructed by the Swiss-Model server (https://swissmodel.expasy.org/) using the crystal structure of GluCl (PDB ID: 3RHW) of *Caenorhabditis elegans* as a template with a high resolution and 64.43% and 55.19% sequence identity to TuGluCl3 and DmGluClα, respectively. The homology models of the two proteins were then evaluated by the SAVES v6.0 (https://saves.mbi.ucla.edu/) and PROSA (https://prosa.services.came.sbg.ac.at/prosa.php) servers. In both models, the amino acid residues in the disallowed regions of the two proteins were less than 10% and the Z-scores related to model energy were also within the range of scores found for experimentally determined proteins of similar size, which indicated that the models were verified to be reasonable in both structure and energy (Supplementary Data 7, Supplementary Fig. 7, Supplementary Fig. 8). The G326E mutant of TuGluCl3 was generated by the Mutate Monomers Module of SYBYL-X 2.1 software (Tripos Inc., USA). The constructed models were then optimized by the Minimize Molecule Module of SYBYL-X 2.1 software using the AMBER7 FF99 force field. The structures of dicofol, bromopropylate, chlorobenzilate, and abamectin compounds were sketched and minimized by the Tripos force field using Gasteiger-Hückel charges with a gradient of 0.005 kcal (mol Å)^-1^ and a maximum iteration number of 10,000. Other parameters were set as default. The molecular dockings were completed by SYBYL-X 2.1 software. The binding pockets of TuGluCl3 WT, TuGluCl3 G326E, and DmGluClα were generated by the Surflex-Dock Module using a residue-based mode and the ligands were docked into the binding pockets. The MOLCAD Module of SYBYL-X 2.1 software was applied to calculate the surface electrostatic and lipophilic potential distributions of the binding sites and ligands to analyze the effects of electrostatic and hydrophobic interactions on the binding of ligands with proteins. The visualization of the docking results was accomplished by PyMOL software (DeLano Scientific LLC, USA).

### Alignment of GluCl TM3 region

An alignment was made of the GluCl TM3 region of various mite families—including Tetranychidae (*Tetranychus urticae, Panonychus citri, Oligonychus coffeae* and *Bryobia spp*.), Tenuipalpidae (*Brevipalpus yotersi*), Phytoseiidae (*Metaseiulus occidentalis*), Demodicidae (*Demodex folliculorum*), Eriophyidae (*Aculops lycopersici* and *Aceria tosichella*), Tarsonemidae (*Acarapis woodii*), Trombiculidae (*Leptotrombidium deliense*) and Varroidea (*Varroa destructor*)—with insect GluCl’s of *Musca domestica* (XP_005183932.3) and *Drosophila melanogaster* (GluClα, NP_001287410.1). *T. urticae* sequences of *TuGluCl1 (tetur02g04080), TuGluCl2 (tetur08g04990), TuGluCl3 (tetur10g03090), TuGluCl4 (tetur22g02450), TuGluCl5 (tetur36g00090)* and *TuGluCl6 (tetur41g00120)* were collected from ORCAE database^92^. GluCl paralogs of *B. yothersi (bryot44g00060, bryot69g00060*) and *A. lycopersici* (*aculy02g05840.1* and *aculy02g13690.1*) were also retrieved from their respective genomes on ORCAE. The protein sequence of TM3 in glutamate-gated chloride channel-like isoform X1 of *V. destructor* was extracted from NCBI (XP_022650816.1). NCBI tblastn searches with the TM3 protein sequence of TuGluCl3 as input was used to identify unannotated paralogs in *P. citri* (three hits with transcribed RNA sequences; GIIF01008809.1, GIIF01001850.1, GIIF01009026.1), *Bryobia spp*. (two hits with genomic sequence reads; SRR15410508), *O. coffeae* (four hits with transcriptomic sequence reads in run id DRR146959), *M. occidentalis* (one hit in whole genome sequencing project; NW_003805141.1), *D. folliculorum* (two hits with genome assemblies; ERR2338209 and ERR2338207), *A. tosichella* (one hit with transcribed RNA sequence; GGYP01007779.1), *A. woodi* (one hit with whole genome sequencing project; BLXL01003973.1) and *L. deliense* (three hits with whole genome sequencing projects; NCKV01013439, NCKV01004263, NCKV01002237). The sequences were aligned using MEGA (version 10.0.5)^95^ using the Clustal W algorithm^96^.

### Statistics and reproducibility

Concentration-response curves, LC_50_-values, slopes, 95% confidence intervals (CIs) and resistance ratios were calculated by probit analysis (POLOplus, LeOra Software, USA). EC_50_, pEC_50_ values and Hill coefficients were calculated by fitting a four-parameter logistic curve (Hill equation) on response data using SigmaPlot software (version 13.0) (Systat Software, USA) and GraphPad Prism software (Dotmatics, USA). Statistical comparison of EC_50_ values was done in R (version 4.2.2) using a Kruskal-Wallis analysis followed by pairwise comparisons using Wilcoxon rank sum exact test with Benjamini-Hochberg adjustment of *p*-values (Supplementary Data 4). *P*-values < 0.05 were considered significant. In figures quantitative data are represented as the mean ± SE with n presented in the figure legends.

### Reporting summary

Further information on research design is available in the Nature Portfolio Reporting Summary linked to this article.

### Supplementary information

Supplementary Figures

Description of Additional Supplementary Files

Supplementary Data 1-7

Reporting Summary

## Data Availability

All the sequence data generated in this study have been submitted to the NCBI Sequence Read Archive (SRA) under BioProject PRJNA990678. Datasets and source data needed to recreate the figures shown in this article have been deposited in Figshare 10.6084/m9.figshare.23668188^29^. Any remaining information can be obtained from the corresponding author upon reasonable request.

## References

[1] March RB (1976). Properties and actions of bridged diphenyl acaricides. Environ. Health Perspect..

[2] Van Leeuwen T, Vontas J, Tsagkarakou A, Dermauw W, Tirry L (2010). Acaricide resistance mechanisms in the two-spotted spider mite *Tetranychus urticae* and other important Acari: a review. Insect Biochem. Mol. Biol..

[3] Baker RT (1985). Resistance of two-spotted mite, *Tetranychus urticae* Koch (Acarina, Tetranychidae), to dicofol in New Zealand - a note. N. Z. J. Exp. Agric..

[4] Van Leeuwen T, Dermauw W (2016). The molecular evolution of xenobiotic metabolism and resistance in chelicerate mites. Annu. Rev. Entomol..

[5] European Parliament, C. of the E. U. *Regulation (EU) 2019/1021 of the European Parliament and of the Council of 20 June 2019 on persistent organic pollutants*. http://data.europa.eu/eli/reg/2019/1021/oj (2019).

[6] Mansouri A (2017). The environmental issues of DDT pollution and bioremediation: a multidisciplinary review. Appl. Biochem. Biotechnol..

[7] Van Den Berg H, Manuweera G, Konradsen F (2017). Global trends in the production and use of DDT for control of malaria and other vector-borne diseases. Malar. J..

[8] Pimentel, D. *Ecological effects of pesticides on non-target species*. (Office of Science and Technology, 1971).

[9] Dittrich V (1975). Acaricide resistance in mites. Z. F.ür. Angew. Entomol..

[10] Fergusson-Kolmes LA, Scott JG, Dennehy TJ (1991). Dicofol resistance in *Tetranychus urticae* (Acari, Tetranychidae) - cross-resistance and pharmacokinetics. J. Econ. Entomol..

[11] Hoyt SC, Harries FH (1961). Laboratory and field studies on orchard-mite resistance to Kelthane. J. Econ. Entomol..

[12] Kim Y, Lee S, Cho J, Park H, Ahn Y (2007). Multiple resistance and biochemical mechanisms of dicofol resistance in *Tetranychus urticae* (Acari: Tetranychidae). J. Asia-Pac. Entomol..

[13] Knowles, C. O. Mechanisms of resistance to acaricides. in *Molecular mechanisms of resistance to agrochemicals* (Springer, Berlin, Heidelberg, 1997). 10.1007/978-3-662-03458-3_3.

[14] Saito, T., Tabata, K. & Kohno, S. Mechanisms of acaricide resistance with emphasis on dicofol. in *Pest resistance to pesticides* (ed. Georghiou, G. P.) (Plenum Press, 1983).

[15] Soderlund DM, Adams PM (1993). Inhibition of octopamine-stimulated adenylate-cyclase activity in two-spotted mites by dicofol and related diphenylcarbinol acaricides. Pestic. Biochem. Physiol..

[16] Sparks TC (2020). Insecticides, biologics and nematicides: updates to IRAC’s mode of action classification - a tool for resistance management. Pestic. Biochem. Physiol..

[17] Van Leeuwen T, Dermauw W, Mavridis K, Vontas J (2020). Significance and interpretation of molecular diagnostics for insecticide resistance management of agricultural pests. Curr. Opin. Insect Sci..

[18] De Rouck, S., İnak, E., Dermauw, W. & Van Leeuwen, T. A review of the molecular mechanisms of acaricide resistance in mites and ticks. *Insect Biochem. Mol. Biol*. 103981 10.1016/j.ibmb.2023.103981 (2023). 10.1016/j.ibmb.2023.103981 37391089

[19] Mota-Sanchez, D. & Wise, J. C. *The arthropod pesticide resistance database*. http://www.pesticideresistance.org (2019). 10.3390/insects15100747 PMC11509053 39452324

[20] Overmeer WPJ, Vanzon AQ, Helle W (1975). Stability of acaricide resistance in spider mite (*Tetranychus urticae*) populations from rose houses. Entomol. Exp. Appl..

[21] Tsagkarakou A (2009). Identification of pyrethroid resistance associated mutations in the para sodium channel of the two-spotted spider mite *Tetranychus urticae* (Acari: Tetranychidae). Insect Mol. Biol..

[22] Van Leeuwen T, Van Pottelberge S, Tirry L (2005). Comparative acaricide susceptibility and detoxifying enzyme activities in field-collected resistant and susceptible strains of *Tetranychus urticae*. Pest Manag. Sci..

[23] Feyereisen R, Dermauw W, Van Leeuwen T (2015). Genotype to phenotype, the molecular and physiological dimensions of resistance in arthropods. Pestic. Biochem. Physiol..

[24] Hatano R, Scott JG, Dennehy TJ (1992). Enhanced activation is the mechanism of negative cross-resistance to chlorpyrifos in the dicofol-ir strain of *Tetranychus urticae* (Acari, Tetranychidae). J. Econ. Entomol..

[25] Tabata K, Saito T (1988). Dicofol detoxification products in resistant citrus red mite *Panonychus-citri* (Mcgregor) and mouse. Appl. Entomol. Zool..

[26] Tabata K, Saito T (1973). Mechanism of dicofol resistance in spider mites II: thin layer chromatographic identification of dicofol metabolites in citrus red mite, *Panonychus citri* McGREGOR. Kyoto Univ. Res. Inf. Repos..

[27] Kurlovs AH (2022). Trans-driven variation in expression is common among detoxification genes in the extreme generalist herbivore *Tetranychus urticae*. PLOS Genet..

[28] Kurlovs AH, Snoeck S, Kosterlitz O, Van Leeuwen T, Clark RM (2019). Trait mapping in diverse arthropods by bulked segregant analysis. Curr. Opin. Insect Sci..

[29] Vandenhole, M. et al. QTL mapping and electrophysiology assays reveal a glutamate-gated chloride channel as the mite-specific target-site of dicofol and other diphenylcarbinol acaricides [Data Set]. *Figshare*10.6084/m9.figshare.23668188 (2023). 10.1038/s42003-023-05488-5 PMC10643420 37957415

[30] Dermauw W (2012). The cys-loop ligand-gated ion channel gene family of *Tetranychus urticae*: implications for acaricide toxicology and a novel mutation associated with abamectin resistance. Insect Biochem. Mol. Biol..

[31] Mermans C, Dermauw W, Geibel S, Van Leeuwen T (2017). A G326E substitution in the glutamate-gated chloride channel 3 (GluCl3) of the two-spotted spider mite *Tetranychus urticae* abolishes the agonistic activity of macrocyclic lactones. Pest Manag. Sci..

[32] Villacis-Perez E (2023). Intraspecific diversity in the mechanisms underlying abamectin resistance in a cosmopolitan pest. Evol. Appl..

[33] Wolstenholme AJ (2012). Glutamate-gated Chloride Channels. J. Biol. Chem..

[34] Xue W (2020). Untangling a Gordian knot: the role of a GluCl3 I321T mutation in abamectin resistance in *Tetranychus urticae*. Pest Manag. Sci..

[35] Kwon DH, Yoon KS, Clark JM, Lee SH (2010). A point mutation in a glutamate-gated chloride channel confers abamectin resistance in the two-spotted spider mite, *Tetranychus urticae* Koch. Insect Mol. Biol..

[36] Gramates LS (2022). FlyBase: a guided tour of highlighted features. Genetics.

[37] Davis P (2022). WormBase in 2022—data, processes, and tools for analyzing *Caenorhabditis elegans*. Genetics.

[38] Sandhu, A., Badal, D., Sheokand, R., Tyagi, S. & Singh, V. Specific collagens maintain the cuticle permeability barrier in *Caenorhabditis elegans*. *Genetics* **217**, iyaa047 (2021). 10.1093/genetics/iyaa047 PMC8045729 33789349

[39] Brown MA, Casida JE (1987). Metabolism of a dicofol impurity alpha-chloro-DDT, but not dicofol or dechlorodicofol, to DDE in mice and a liver microsomal system. Xenobiotica.

[40] Wang X (2017). Mutations on M3 helix of *Plutella xylostella* glutamate-gated chloride channel confer unequal resistance to abamectin by two different mechanisms. Insect Biochem. Mol. Biol..

[41] Beasley, V. R. Direct and indirect effects of environmental contaminants on amphibians. in *Reference Module in Earth Systems and Environmental Sciences* (Elsevier, 2020). 10.1016/B978-0-12-409548-9.11274-6.

[42] Al-Rubae AY, Knowles CO (1972). Metabolism of chloropropylate and bromopropylate acaricides by twospotted spider mites and house flies. J. Econ. Entomol..

[43] Hibbs RE, Gouaux E (2011). Principles of activation and permeation in an anion-selective Cys-loop receptor. Nature.

[44] Atif M, Estrada-Mondragon A, Nguyen B, Lynch JW, Keramidas A (2017). Effects of glutamate and ivermectin on single glutamate-gated chloride channels of the parasitic nematode *H. contortus*. PLOS Pathog..

[45] Royalty RN, Perring TM (1987). Comparative toxicity of acaricides to *Aculops lycopersici* and *Homeopronematus anconai* (Acari: Eriophyidae, Tydeidae). J. Econ. Entomol..

[46] Van Laere O, Ifantidis M, De Wael L (1983). Dicofol-räuchern von honigbienen zur bekämpfung der milbe *Varroa jacobsoni*. Apidologie.

[47] O’Reilly AO (2014). Predictive 3D modelling of the interactions of pyrethroids with the voltage-gated sodium channels of ticks and mites: Interactions of pyrethroids with voltage-gated sodium channels. Pest Manag. Sci..

[48] Menzel DB, Miskus R, Hoskins WM, Smith SM (1961). Metabolism of C14-labeled DDT in larvae, pupae, and adults of *Drosophila melanogaster*. J. Econ. Entomol..

[49] Tsukamoto M (1959). Metabolic fate of DDT in *Drosophila melanogaster*. Identification of a non-DDE metabolite. Sci. Pest Control.

[50] Amichot M (2004). Point mutations associated with insecticide resistance in the *Drosophila* cytochrome P450 *Cyp6a2* enable DDT metabolism: DDT metabolism by a mutant CYP6A2. Eur. J. Biochem..

[51] Cùany A (1990). Characterization of microsomal oxidative activities in a wild-type and in a DDT resistant strain of *Drosophila melanogaster*. Pestic. Biochem. Physiol..

[52] Morello A (1964). Role of DDT-hydroxylation in Resistance. Nature.

[53] Rolofson, G. L. *In vivo studies of suspected mechanisms of DDT-resistance in Blatella germanica (L.)*. (Virginia Polytechnic Institute, 1968).

[54] Sawicki RM (1978). Unusual response of DDT-resistant houseflies to carbinol analogs of DDT. Nature.

[55] Seiber, N. J. & Kleinschmidt, L. Environmental transport and fate. in *Hayes’ Handbook of Pesticide Toxicology* (ed. Krieger, R.) 1219–1227 (Academic Press, 2010). 10.1016/B978-0-12-374367-1.00057-4.

[56] Al-Jboory IJ, Jumida RE, Al-Sammarie AI (2004). Cross resistance of bromopropylate in the two spotted spider mite *Tetranychus urticae* Koch (Acari: Tetranychidae). Univ. Aden J. Nat. Appl. Sci..

[57] Alves EB, Omoto C, Franco CR (2000). Cross-resistance between dicofol and other acaricides in *Brevipalpus phoenicis* (Acari: Tenuipalpidae). Soc. Entomol.ógica Bras..

[58] Horowitz AR, Weintraub PG, Ishaaya I (1998). Status of pesticide resistance in arthropod pests in Israel. Phytoparasitica.

[59] Mansour FA, Plaut HN (1979). Effectiveness of various acaricides against resistant and susceptible carmine spider mites. Phytoparasitica.

[60] Zhang, S. Acaricide composition containing bromopropylate and avermectin with synergistic action. (2008).

[61] Clark JM, Scott JG, Campos F, Bloomquist JR (1995). Resistance to avermectins: extent, mechanisms, and management implications. Annu. Rev. Entomol..

[62] Jeppson, L. R. New acaricide for citrus mites - chlorobenzilate formulations have low toxicity to warm-blooded animals but in tests gave effective control of mites on citrus. *Calif. Agric*. 10.3733/ca.v009n06p11 (1955).

[63] Dagli F, Tunc I (2001). Dicofol resistance in *Tetranychus cinnabarinus*: resistance and stability of resistance in populations from Antalya, Turkey. Pest Manag. Sci..

[64] Kim G-H, Song C, Chang BY, Park N-J, Cho K-Y (1995). Stability of dicofol resistance of the two-spotted spider mite, *Tetranychus urticae* Koch (Acarina: Tetranychidae). Korean J. Appl. Entomol..

[65] Dennehy, T. J., Nyrop, J. P. & Martinson, T. E. Characterization and exploitation of instability of spider-mite resistance to acaricides. in *Acs Sym Ser* (eds. Green, M. B., Lebaron, H. M. & Moberg, W. K.) vol. 421 77–91 (1990).

[66] Inoue, K. Relationship between dicofol resistance and fitness in the citrus red mite, *Panonychus citri* (McG.). *J. Pesticide Sci* 165–175 (1980).

[67] Bajda S (2018). Fitness costs of key point mutations that underlie acaricide target-site resistance in the two-spotted spider mite *Tetranychus urticae*. Evol. Appl..

[68] Dennehy TJ, Granett J (1984). Spider mite resistance to dicofol in San Joaquin Valley cotton - interspecific and intraspecific variability in susceptibility of 3 species of *Tetranychus* (Acari, Tetranychidae). J. Econ. Entomol..

[69] Inoue K (1979). The change of susceptibility of mite population to dicofol and genetic analysis of dicofol-resistance in the Citrus Red Mite, *Panonychus citri* (McG.): studies on acaricide resistance in the Citrus Red Mite (Part I). J. Pestic. Sci..

[70] Kim G-H, Song C, Park N-J, Cho K-Y (1994). Inheritance if resistance in dicofol-selected strain of the two-spotted spider mite, *Tetranychus urticae* Koch (Acarina: Tetranychidae), and its cross resistance. Korean J. Appl. Entomol..

[71] Martinson TE, Dennehy TJ, Nyrop JP, Reissig WH (1991). Field-measurements of selection for 2-spotted spider-mite (Acari, Tetranychidae) resistance to dicofol in apple orchards. J. Econ. Entomol..

[72] Ghosh R, Andersen EC, Shapiro JA, Gerke JP, Kruglyak L (2012). Natural variation in a chloride channel subunit confers Avermectin resistance in *C. elegans*. Science.

[73] Riga M (2017). The relative contribution of target-site mutations in complex acaricide resistant phenotypes as assessed by marker assisted backcrossing in *Tetranychus urticae*. Sci. Rep..

[74] Wang X (2016). A point mutation in the glutamate-gated chloride channel of *Plutella xylostella* is associated with resistance to abamectin: A mutated *PxGluCl* confers abamectin resistance. Insect Mol. Biol..

[75] Ludmerer SW (2002). Ivermectin and Nodulisporic Acid Receptors in *Drosophila melanogaster* Contain Both γ-Aminobutyric Acid-Gated Rdl and Glutamate-Gated GluClα Chloride Channel Subunits. Biochemistry.

[76] Papapostolou KM (2022). Over-expression in cis of the midgut P450 CYP392A16 contributes to abamectin resistance in *Tetranychus urticae*. Insect Biochem. Mol. Biol..

[77] Riga M (2014). Abamectin is metabolized by CYP392A16, a cytochrome P450 associated with high levels of acaricide resistance in Tetranychus urticae. Insect Biochem. Mol. Biol..

[78] Kono, S., Saito, T. & Miyata, T. Mechanism of Resistance to Dicofol in the Two-Spotted Spider Mite, Tetranychus urticae KOCH (Acarina: Tetranychidae). *Jap. J. appl. Ent. Zool*. 101–107 (1981).

[79] Thiel A, Guth S, Böhm S, Eisenbrand G (2011). Dicofol degradation to p,p′-dichlorobenzophenone – A potential antiandrogen. Toxicology.

[80] Khajehali J, Van Nieuwenhuyse P, Demaeght P, Tirry L, Van Leeuwen T (2011). Acaricide resistance and resistance mechanisms in *Tetranychus urticae* populations from rose greenhouses in the Netherlands. Pest Manag. Sci..

[81] Van Laecke K, Degheele D (1993). Effect of insecticide—synergist combinations on the survival of *Spodoptera exigua*. Pestic. Sci..

[82] R. Core Team. *R: a language and environment for statistical computing*. (2022).

[83] Grbic M (2011). The genome of *Tetranychus urticae* reveals herbivorous pest adaptations. Nature.

[84] Wybouw N (2019). Long-term population studies uncover the genome structure and genetic basis of xenobiotic and host plant adaptation in the herbivore *Tetranychus urticae*. Genetics.

[85] Li H, Durbin R (2009). Fast and accurate short read alignment with Burrows-Wheeler transform. Bioinformatics.

[86] Li H (2009). The Sequence Alignment/Map format and SAMtools. Bioinformatics.

[87] McKenna A (2010). The Genome Analysis Toolkit: a MapReduce framework for analyzing next-generation DNA sequencing data. Genome Res..

[88] Hahne, F. & Ivanek, R. Visualizing genomic data using Gviz and Bioconductor. in *Statistical Genomics* (eds. Mathé, E. & Davis, S.) vol. 1418 335–351 (Springer New York, 2016). 10.1007/978-1-4939-3578-9_16 27008022

[89] Snoeck S (2019). High-resolution QTL mapping in *Tetranychus urticae* reveals acaricide-specific responses and common target-site resistance after selection by different METI-I acaricides. Insect Biochem. Mol. Biol..

[90] Wickham, H. *ggplot2*. (2009).

[91] Cingolani P (2012). A program for annotating and predicting the effects of single nucleotide polymorphisms, SnpEff: SNPs in the genome of *Drosophila melanogaster strain* w1118; iso-2; iso-3. Fly. (Austin).

[92] Sterck L, Billiau K, Abeel T, Rouze P, Van de Peer Y (2012). ORCAE: online resource for community annotation of eukaryotes. Nat. Methods.

[93] Cully DF, Paress PS, Liu KK, Schaeffer JM, Arena JP (1996). Identification of a *Drosophila melanogaster* glutamate-gated chloride channel sensitive to the antiparasitic agent avermectin. J. Biol. Chem..

[94] Mermans, C., Dermauw, W., Geibel, S. & Van Leeuwen, T. Activity, selection response and molecular mode of action of the isoxazoline afoxolaner in *Tetranychus urticae*. *Pest Manag. Sci*. ps.7187 10.1002/ps.7187 (2022). 10.1002/ps.7187 36116012

[95] Kumar S, Stecher G, Li M, Knyaz C, Tamura K (2018). MEGA X: Molecular Evolutionairy Genetics Analysis across Computing Platforms. Mol. Biol. Evol..

[96] Larkin MA (2007). Clustal W and Clustal X version 2.0. Bioinformatics.

