# Supplementary material for: A glutamate-gated chloride channel as the mite-specific target-site of dicofol and other diphenylcarbinol acaricides
Source: Commun Biol. 2023 Nov 13;6:1160. doi: 10.1038/s42003-023-05488-5 (PMC10643420; doi:10.1038/s42003-023-05488-5)
Supplement: Supplementary file 1 — Supplementary Figures [file 42003_2023_5488_MOESM1_ESM.docx]

**Supplementary figures**

**Supplementary Figure 1**. Deviations in allele frequencies of dicofol (DIC) selected and unselected (control, CON) populations.

**
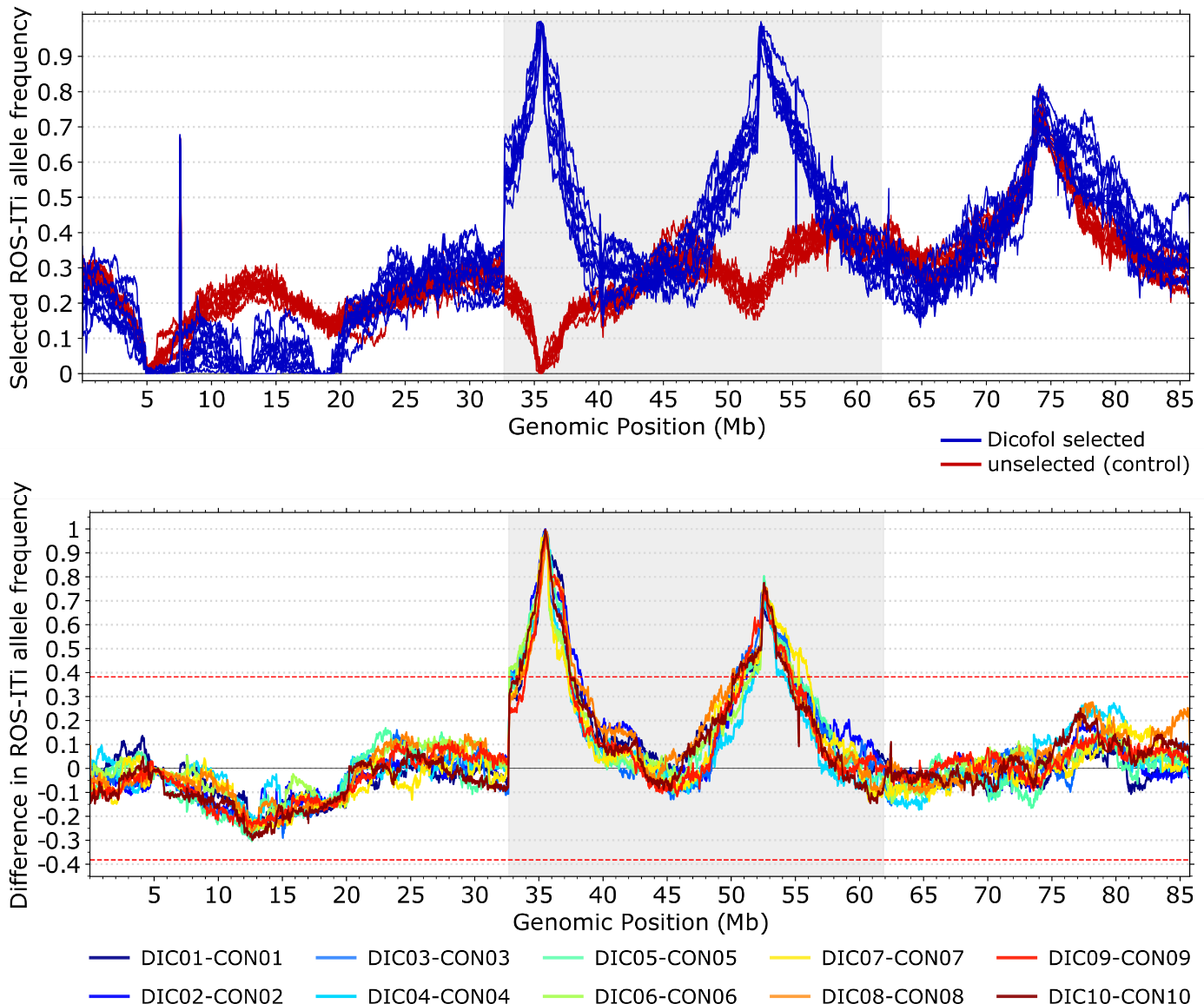
**

**Supplementary Figure 2**. Dicofol, chlorobenzilate, bromopropylate and abamectin dose–response curves for the activation of homomeric TuGluCl3 expressed in *Xenopus* oocytes. The response of each oocyte to the specific compound was normalized and expressed as a percentage of the EC_50_ L-glutamic acid response. Error bars indicate SEM (n=6). Abamectin dose-response data was previously published in Mermans et al. (2017)^33^.

**
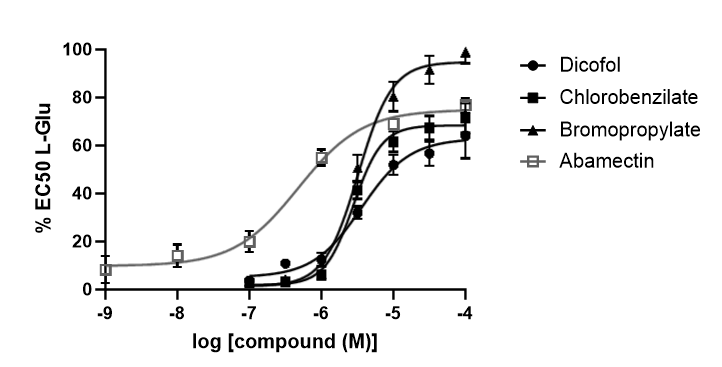
**

**Supplementary Figure 3**. The binding pattern of abamectin components AVM B1a (left) and AVM B1b (right) in the active site of TuGluCl3 WT (upper panel), TuGluCl3 G326E (middle panel) or DmGluClα (lower panel). The protein is shown as cartoon. The hydrogen bond and π-π stacking are shown as blue and purple dashes, respectively.

**
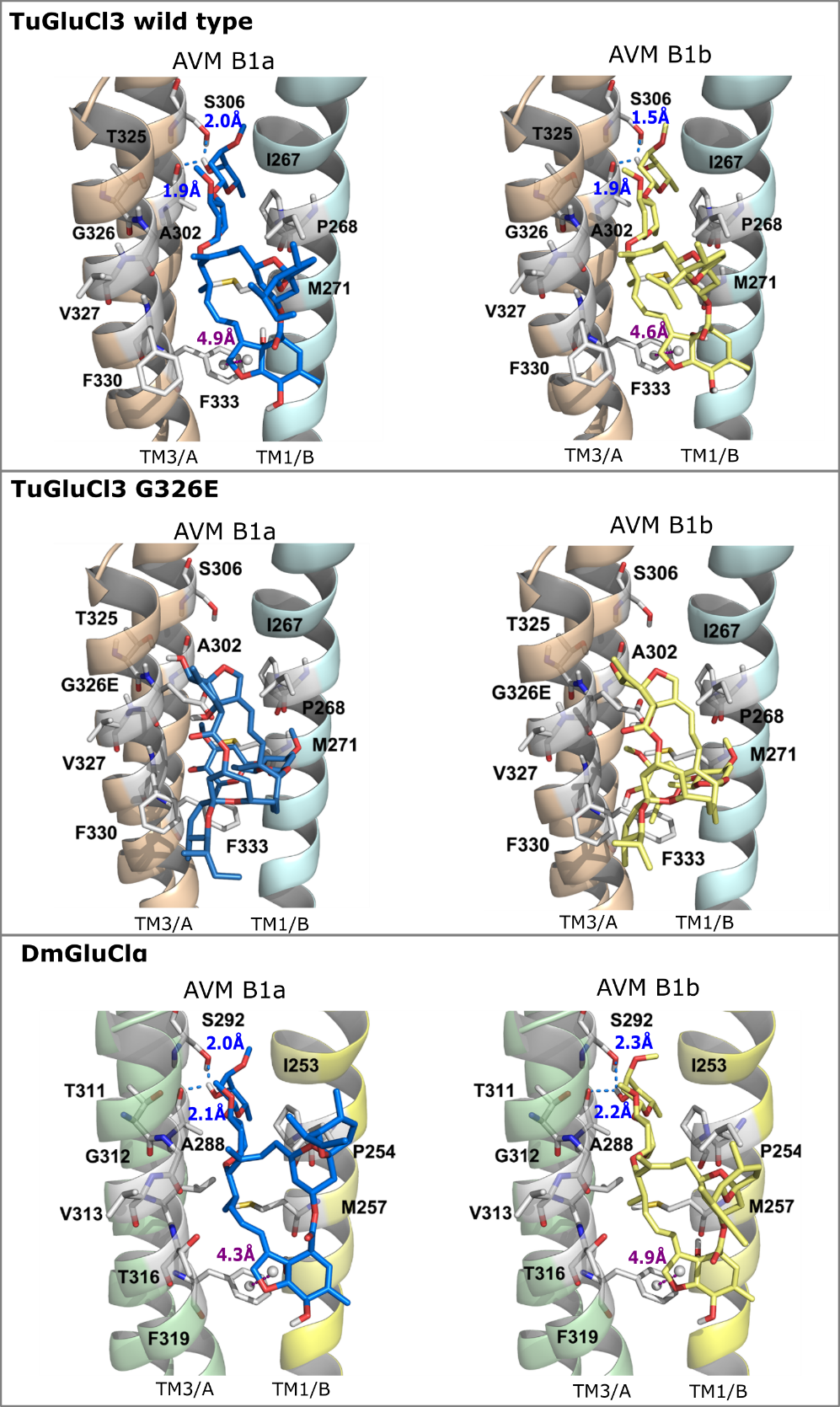
**

**Supplementary Figure 4**. The binding pattern of bromopropylate and chlorobenzilate in the active site of TuGluCl3 WT and TuGluCl3 G326E. The protein is shown as cartoon. The hydrogen bond is shown as a yellow dash.

**
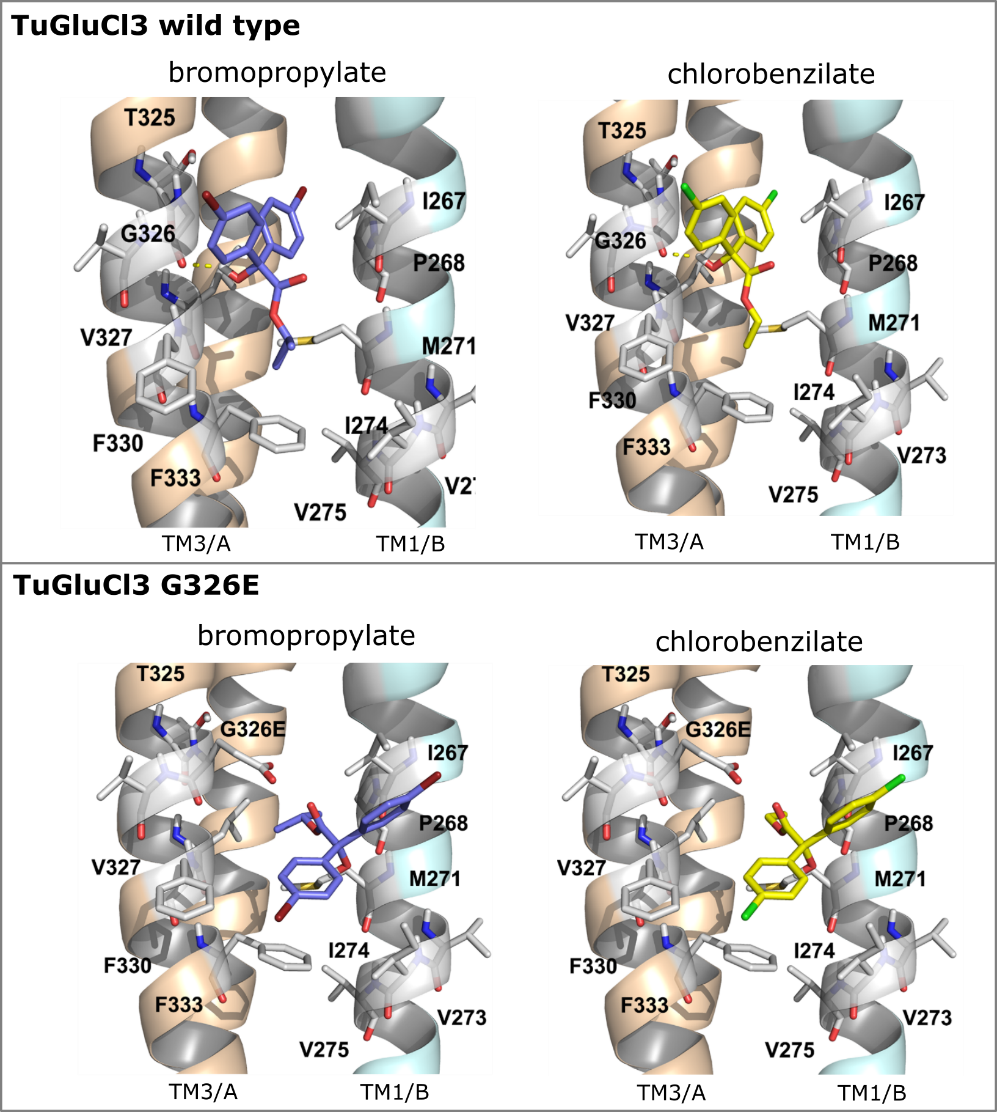
**

**Supplementary Figure 5**. The binding pattern of DDT in the active site of TuGluCl3 WT.**
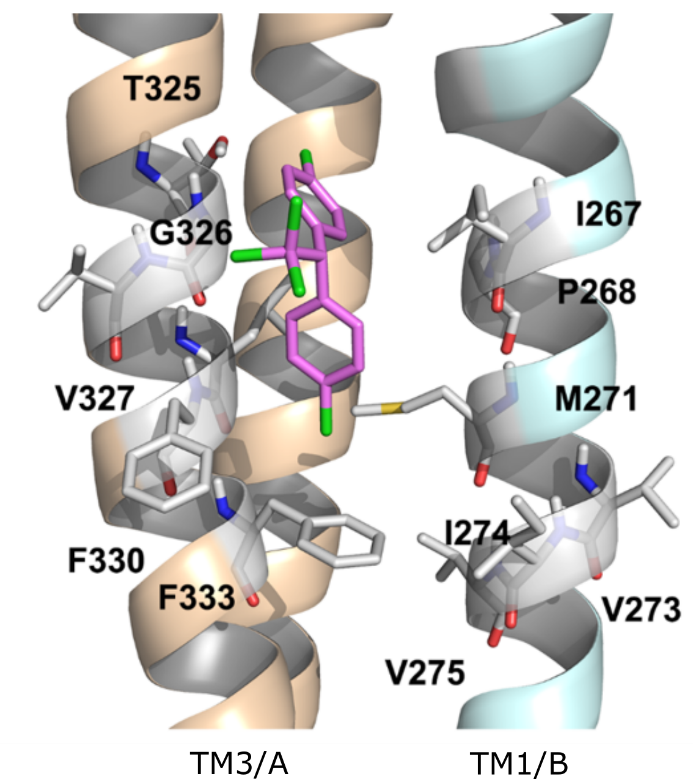
**

**Supplementary Figure 6**. Alignment of the GluCl TM3 region of various mite families - including Tetranychidae (*Tetranychus urticae, Panonychus citri, Oligonychus coffeae* and *Bryobia spp.*), Tenuipalpidae (*Brevipalpus yothersi*), Phytoseiidae (*Metaseiulus occidentalis*), Demodicidae (*Demodex folliculorum*), Eriophyidae (*Aculops lycopersici* and *Aceria tosichella*), Tarsonemidae (*Acarapis woodii*), Trombiculidae (*Leptotrombidium deliense*) and Varroidae (*Varroa destructor*) - with insect GluCls of *Musca domestica* and *Drosophila melanogaster* (GluClα). The variation at location F330 of TuGluCl3 is indicated with a red rectangle.

**
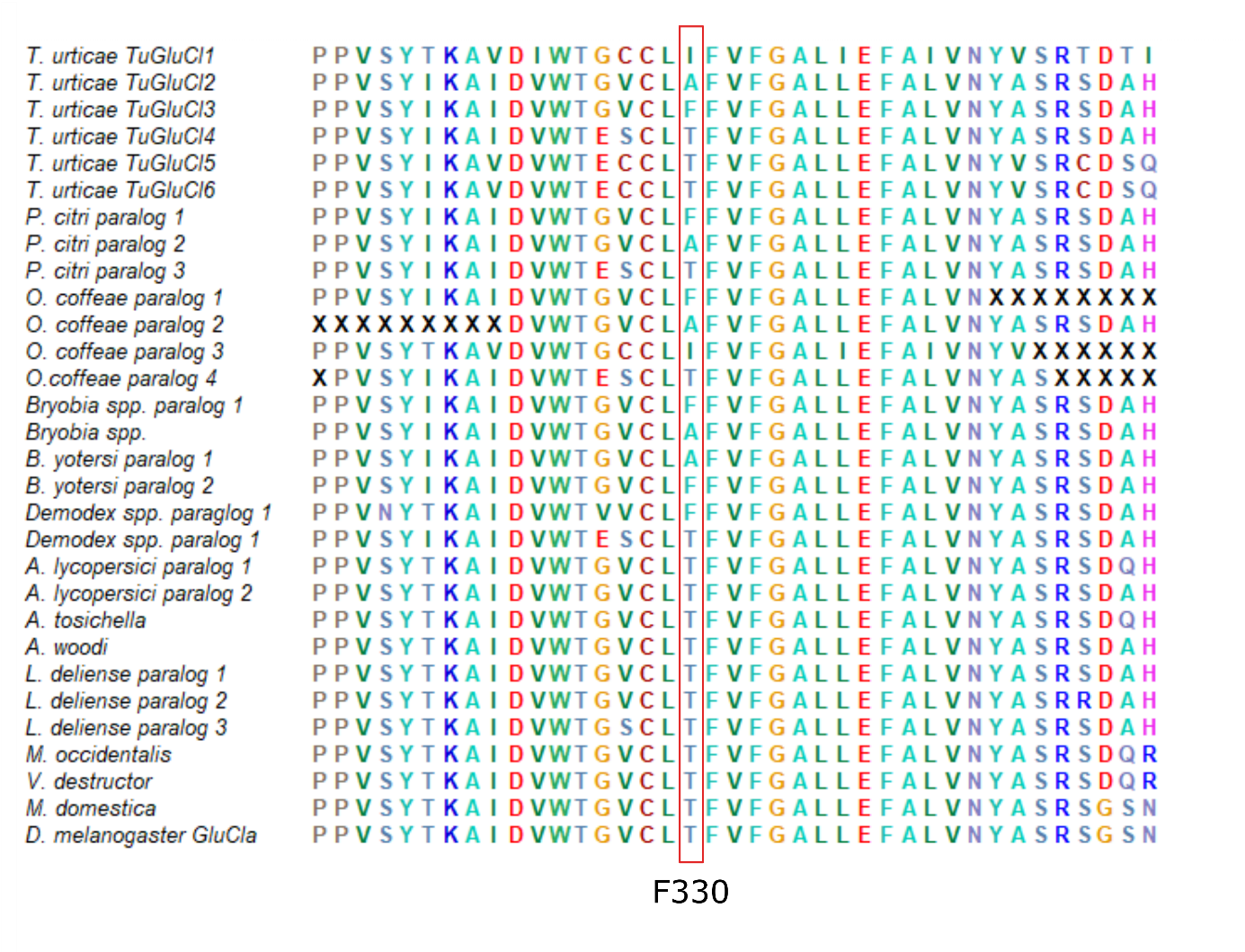
**

**Supplementary Figure 7**. The evaluation results of Ramachandran plot (a) and Z-score distribution plot (b) of the modeled structure of TuGluCl3.


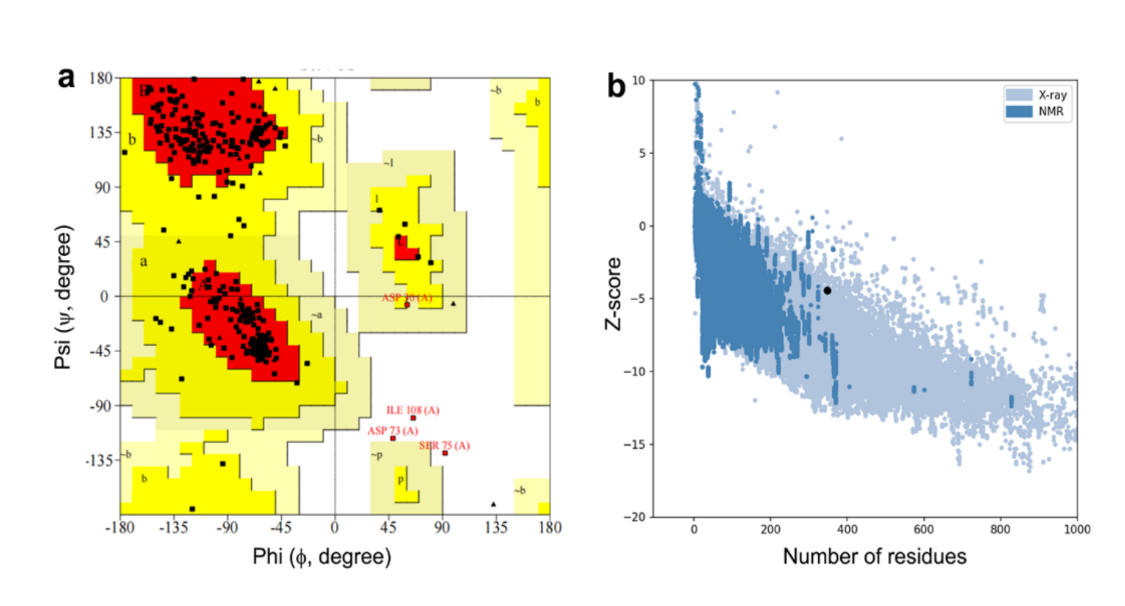


**Supplementary Figure 8**. The evaluation results of Ramachandran plot (a) and Z-score distribution plot (b) of the modeled structure of DmGluClα.

**
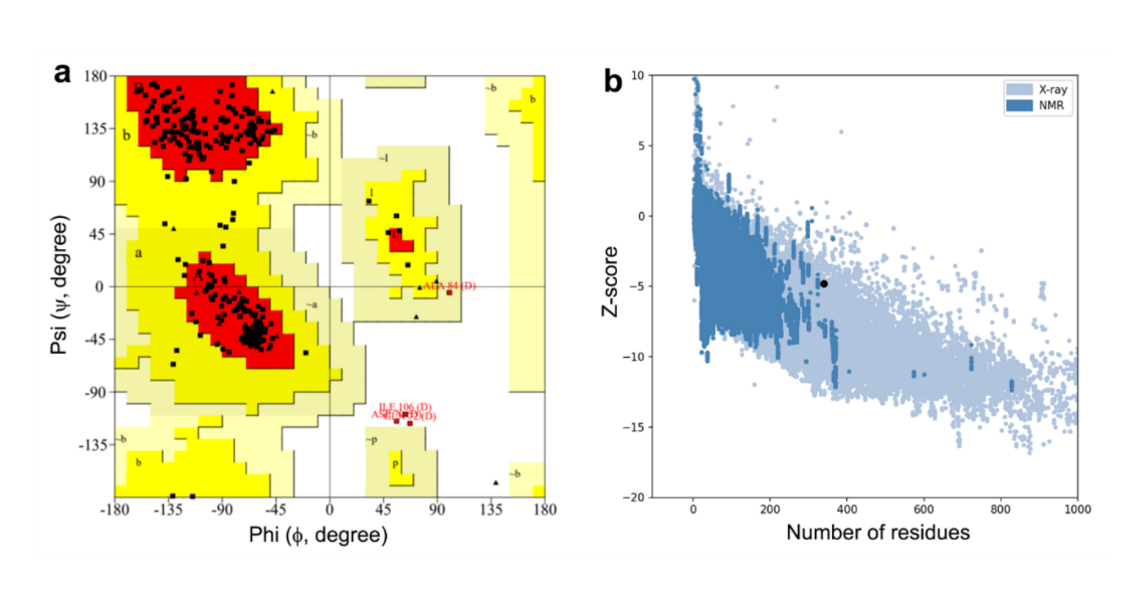
**
