# Supplementary material for: A glutamate-gated chloride channel as the mite-specific target-site of dicofol and other diphenylcarbinol acaricides
Source: Commun Biol. 2023 Nov 13;6:1160. doi: 10.1038/s42003-023-05488-5 (PMC10643420; doi:10.1038/s42003-023-05488-5)
Supplement: Supplementary file 2 — Description of Additional Supplementary Files [file 42003_2023_5488_MOESM2_ESM.pdf]

Description of Additional Supplementary Files

File name: Supplementary Data 1
Description: Genes covered by QTL1 (Chr. 2, 2.862 Mb ± 500 kb) of BSAdicofol.

File name: Supplementary Data 2
Description: Allele frequency of enriched resistance mutations in TuGluCl1 and TuGluCl3 in all replicates and parental strains.

File name: Supplementary Data 3
Description: Genes covered by QTL2 (Chr.2, 19.907 Mb ± 500 kb) of BSAdicofol.

File name: Supplementary Data 4
Description: Statistical comparison of EC50 values of compounds acting on TuGluCl3 WT by means of a Kruskal-Wallis analysis followed by pairwise comparisons using Wilcoxon rank sum exact test with Benjamini-Hochberg adjustment of p-values.

File name: Supplementary Data 5
Description: Potentiation data for compounds acting on TuGluCl3 WT.

File name: Supplementary Data 6
Description: DmGluClα constructs used in TEVC assays.

File name: Supplementary Data 7
Description: The statistical evaluation of the TuGluCl3 and DmGluClα models.
